# Mutual impact of clinically translatable near-infrared dyes on photoacoustic image contrast and *in vitro* photodynamic therapy efficacy

**DOI:** 10.1117/1.JBO.25.6.063808

**Published:** 2020-02-28

**Authors:** Ljubica Z. Petrovic, Marvin Xavierselvan, Maju Kuriakose, Michael D. Kennedy, Christopher D. Nguyen, Julian J. Batt, Kelsey B. Detels, Srivalleesha Mallidi

**Affiliations:** Tufts University, Department of Biomedical Engineering, Medford, Massachusetts, United States

**Keywords:** photoacoustic imaging, photodynamic therapy, photosensitizer, image-guided therapy, indocyanine green, benzoporphyrin derivative

## Abstract

Photodynamic therapy (PDT), a spatially localized phototoxic therapy that involves irradiation of a photosensitizer (PS) with specific wavelengths of light, has shown exceptional promise in impacting cancer treatment outcomes, particularly oral cancer. To reduce PDT outcome variability, attempts toward image-guided personalized PDT are being pursued by monitoring PS uptake either via fluorescence or photoacoustic imaging (PAI), a nonionizing modality dependent on optical absorption properties of the tissue. PAI-guided PDT requires a near-infrared contrast agent for deep tissue imaging with minimal photobleaching effect. We evaluate the impact of PDT agent, benzoporphyrin derivative (BPD), on PAI agent indocyanine green (ICG) and vice versa, given that they have different optical absorption properties and singlet oxygen quantum yields for PDT. Specifically, we demonstrate in two oral squamous cell carcinoma lines (FaDu and SCC4) that ICG has minimal effect on BPD PDT efficacy when irradiated with either a continuous or pulsed laser. Furthermore, the impact of BPD on ICG photodegradation was monitored with PAI in tissue-mimicking phantoms. These studies inform us that the combination of BPD and ICG can be utilized for PAI-guided PDT. However, researchers need to consider the photodegradation effects of ICG in the presence of BPD when designing their drug delivery strategies for PAI-guided PDT.

## 1 Introduction

The past few decades have seen exponential growth in the utility of light for diagnosis and therapy of cancer. Specifically, in the cancer therapeutics realm, photodynamic therapy (PDT) has shown exceptional promise in impacting outcomes, due to spatially and temporally localized treatment with less systemic toxicity.^1^^–^^5^ PDT is a light-based therapy that uses a photosensitizer (PS) to generate cytotoxic reactive molecular species such as the singlet oxygen molecules to target and kill cancer cells. Clinically, PDT’s worth as a mainstream therapy is currently being explored for various skin cancers, head and neck cancer, brain tumors, pulmonary and pleural mesothelial cancer, gastroenterological cancer, and urological disease, as well as for oral cancer.^6^^,^^7^ Recently, the efficacy of PDT was demonstrated in oral cancer patients with early stage lesions. A single session of PDT treatment resulted in negative biopsies and excellent healing of the mucosa.^8^ Despite promising results, one of the main hurdles for PDT in cancer has been variations in treatment outcome due to intertumor and intratumor heterogeneity in PS uptake due to irregular tumor vascular structure.^5^^,^^9^^,^^10^ As the PS molecule is inherently a theranostic agent, its fluorescence properties have traditionally been utilized to monitor uptake and photobleaching to predict PDT response and reduce the variability in the treatment outcome.^11^^,^^12^ However, due to the inability of wide field fluorescence imaging technique to provide three-dimensional images, a comprehensive picture of PS uptake cannot be obtained and there is a need for imaging modalities, such as photoacoustic imaging (PAI), to obtain the heterogeneous distribution of the PS in the tumor.

In PAI, the delivered nanosecond pulsed light energy is absorbed by the dye and converted into heat, leading to thermoelastic expansion and subsequent ultrasound wave emission unlike the radiative relaxation of the excited dye molecule in fluorescence imaging.^13^^–^^16^ Primarily, the PAI contrast depends on the optical absorption properties of the dye that enables it to monitor dye uptake^17^^,^^18^ and dye–environment interactions.^19^^,^^20^ In the near-infrared (NIR) optical window range, indocyanine green (ICG) and IRdye800CW are the only U.S. Food and Drug Administration (FDA)-approved dyes for imaging that can provide photoacoustic (PA) contrast. ICG is used as a PA contrast agent either in its free form or associated with different types of nanoparticles.^21^ ICG is not a good PS due to its meager singlet oxygen quantum yield (0.10 to 0.20),^22^^,^^23^ but it is an excellent contrast agent. On the other hand, benzoporphyrin derivative (BPD) is the only FDA-approved NIR PS with high singlet oxygen quantum yield of 0.77^24^ and is not conditioned to be a good PAI agent. Despite being categorized as theranostic agents, neither BPD nor ICG can individually be used successfully for both PDT and PAI simultaneously. Furthermore, the pulsed irradiation utilized for PAI could also be used to excite BPD to generate singlet oxygen and the resultant therapeutic effect is on par with continuous wave (CW) irradiation.^25^ Hence, several avenues are being explored to design entities that can be both excellent PAI and PDT agents, such as conjugating PS with gold nanovesicles that are good PA contrast agents^26^ or encapsulating both PA and PDT agents within a nanoentity, such as liposomes.^27^^–^^31^ Specifically in the latter case, it becomes critical to evaluate the interactions between the optical dyes, such as BPD and ICG, prior to extensive exploration on the nanoformulations. Loading the nanoentities with high concentrations of BPD and ICG for better signal-to-noise ratio or higher PS delivery can lead to unstable nanoparticles, quenching, and aggregation effects. While the combination of ICG and BPD is untried in a nanoformulation, in this study, we explore for the first time the mutual effects of ICG on BPD’s phototoxicity and BPD’s oxidation effect on ICG’s PA signal-generation capabilities. We also perform studies on two oral cancer cell lines to show the phototoxic effect of BPD when irradiated with a CW laser generally used for PDT or a nanosecond pulsed laser light that is utilized for PAI. These studies inform us that BPD and ICG combination can be utilized for PAI-guided PDT, and it points the need to consider the mutual impact when designing drug delivery strategies.

## 2 Materials and Methods

### 2.1 Cell Culture

FaDu (hypopharyngeal squamous cell carcinoma) and SCC4 (tongue squamous cell carcinoma) cell lines were purchased from American Type Culture Collection. FaDu and SCC4 were cultured using Eagle’s minimum essential medium (EMEM) and Dulbecco’s modified Eagle’s medium (DMEM) media, respectively. The media was supplemented with fetal bovine serum and antibiotics. Cells were grown in T75 flasks and maintained in a standard cell culture environment.

### 2.2 Photodynamic Efficacy of Benzoporphyrin Derivative in the Presence of Indocyanine Green

Post trypsinization, cells were counted and plated on a 24-well plate at a density of 20,000 cells per well. One day later, fresh media containing BPD (Benzoporphyrin derivative monoacid ring A, Verteporfin, 718.794  g/mol, Sigma-Aldrich) and ICG (774.98  g/mol, U.S. Pharmacopeia) in varying ratios (BPD:ICG concentrations—0.25:0, 0.25:0.0625, 0.25:0.25, 0.25:0.5, 0.25:1, and 0:1  μM, respectively) was added to the cells and incubated for 1 h. The concentration and incubation period was chosen for minimal dark toxicity and maximal dye uptake based on previously reported studies.^18^^,^^32^^–^^34^ After the old media was replaced with fresh media, the cells were irradiated with a 690 nm CW laser (HPD 7401 Laser Source) at an irradiance of $50\;\mathrm{mW}/{\mathrm{cm}}^{2}$ and energy densities of 1, 5, and $10\;J/{\mathrm{cm}}^{2}$. To evaluate if PAI by itself could cause cell damage with BPD or ICG, in another set of experiments, the cells were treated with 690 and 800 nm pulsed laser (Optical Parameter Oscillator Laser from Opotek, Phocus BENCHTOP, 690 to 950 nm) at an energy of 5  mJ/pulse for 200, 1000, and 2000 pulses to match the irradiation time equivalent to the CW dose. Cell viability was calculated 24 h post treatment using Vybrant MTT Cell Proliferation Assay Kit (#V13154, Thermofisher). Six experimental repeats were performed.

### 2.3 Benzoporphyrin Derivative and Indocyanine Green Interactions in Solutions

For quantifying the decrease in absorbance of ICG due to PDT, we devised an experiment where solutions of BPD and ICG at various ratios in dimethyl sulfoxide (DMSO) were irradiated with a CW laser at 690 nm or a nanosecond pulsed laser operating either at 690 or 800 nm wavelength. The CW laser irradiance was $50\;\mathrm{mW}/{\mathrm{cm}}^{2}$ and solutions received a dose of 5, 10, and $20\;J/{\mathrm{cm}}^{2}$. The pulsed laser energy was 5  mJ/pulse and solutions were irradiated with 1000, 2000, and 4000 pulses to match the CW laser dose. Prior to and after the irradiation, absorption spectra of the solutions were recorded using a Synergy H1 microplate reader (BioTek).

### 2.4 Photoacoustic Imaging of Tissue-Mimicking Phantoms

**Fig. 1:**
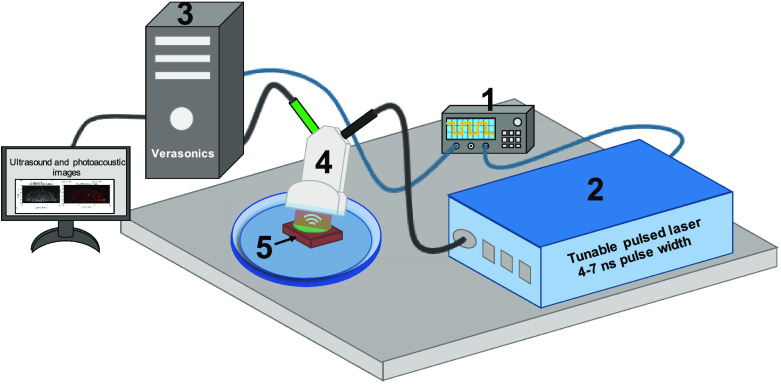
Schematic representation of the imaging setup to obtain US and PA images of the tissue-mimicking phantoms with a high-frequency linear array transducer. (1) Function generator, (2) tunable pulsed laser (4 to 7 ns pulse width), (3) Verasonics Vantage 256 high-frequency US platform, (4) combined US and PA transducer, and (5) tissue-mimicking phantom (photograph of the phantom provided in Fig. S1 in the Supplementary Material)

To prepare tissue-mimicking phantoms, FaDu cells were incubated with EMEM media containing BPD (1 h) and ICG (24 h) in various ratios (BPD:ICG concentrations—0:5, 5:0, 5:5, and 0.25:1  μM). The cells were washed with phosphate-buffered saline and collected using trypsin. For each condition, $20\times 10^{6}$ cells were mixed with 400  μL of 8% (w/v) gelatin and plated on a plain gelatin bed inside a silicone rubber spacer (13 mm diameter) in a 60-mm Petri dish. The photographs of the phantom are provided in Fig. S1 in the Supplementary Material. PAI and ultrasound (US) images were acquired using a VisualSonics LZ250 optical fiber bundle-coupled 15 MHz ultrasound transducer (bandwidth = 13 to 24 MHz, number of elements = 256, and aperture = 22.5 mm) connected to the Verasonics HF Vantage 256 system. A Q-switched, wavelength tunable laser operated at 10 Hz pulse repetition rate and 4 to 7 ns pulse width was used as the coherent light source. Light was delivered through a fiber-optic bundle focused at around 11-mm depth away from the transducer. The Vantage system and the Opotek laser were triggered using a Rigol DG 4102 signal generator at single polarity 5 V, 10 Hz square wave with 20% duty cycle (10 ms pulse width). The graphical representation of our imaging set up is shown in Fig. 1, where the tissue-mimicking sample was in the transducer imaging plane inside a Petri dish filled with deionized water for acoustic coupling between the transducer and the sample. To understand the ICG degradation in different sample combinations (with or without BPD) due to pulsed light irradiation, samples were irradiated with each wavelength (690 or 800 nm) for up to 900 s at a rate of 10 pulses per second and $15\;\mathrm{mJ}/{\mathrm{cm}}^{2}$ per pulse. PA monitoring was performed at 690 or 800 nm at every 150 s intervals. All experiments were done at ambient room temperature conditions and the data were processed using customized MATLAB codes. PA images were captured at a frame rate of 10 Hz and the final image displayed in Figs. 6(a)–6(i) was obtained by averaging four consecutive images. The images generated had lateral and axial pixel dimensions of 88 and 44  μm, respectively. The PA images were compensated for the difference in laser output energy at the two wavelengths (690 and 800 nm), and the background signal (captured at 900 nm, wavelength where BPD and ICG have negligible absorption) was subtracted from the resultant image. The PA signal amplitudes from the different tissue-mimicking phantoms are displayed as a function of time in Figs. 6(j) and 6(k).

## 3 Results and Discussion

### 3.1 Indocyanine Green Has Minimal Impact on the Photodynamic Efficacy of Benzoporphyrin Derivative *In Vitro*

**Fig. 2:**
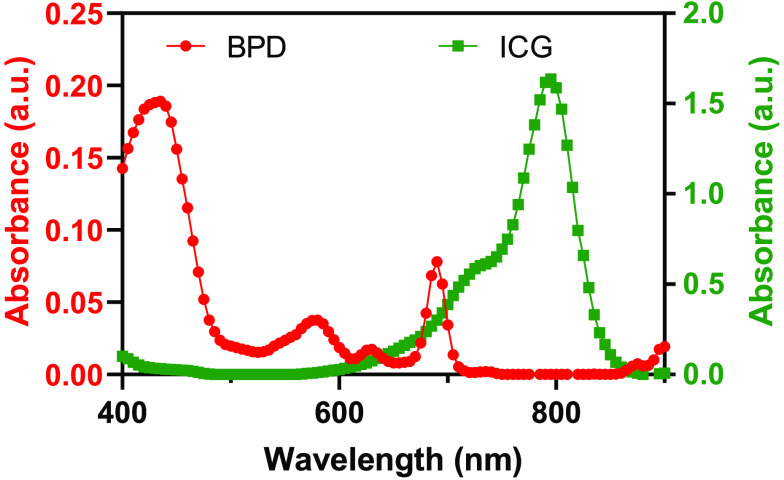
Absorption spectra of BPD and ICG (5  μM concentration each). ICG has lower absorption near the Soret band of BPD, whereas BPD has lower absorption at 800 nm.

ICG has an absorption peak at around 800 nm with broader absorption in the 675 to 845 nm range at ∼13.5% of the peak absorbance (Fig. 2, green line). BPD has two absorption peaks, namely the Soret band at 420 nm and the Q band at 690 nm, respectively (Fig. 2, red line). ICG has low absorption in the Soret band region of BPD, whereas BPD has low absorption at wavelengths greater than 720 nm. It can be seen in Fig. 2 that both ICG and BPD have significant absorption at 690 nm, which makes it necessary to study the interactions when they are used in combination for PAI-guided PDT.

**Fig. 3:**
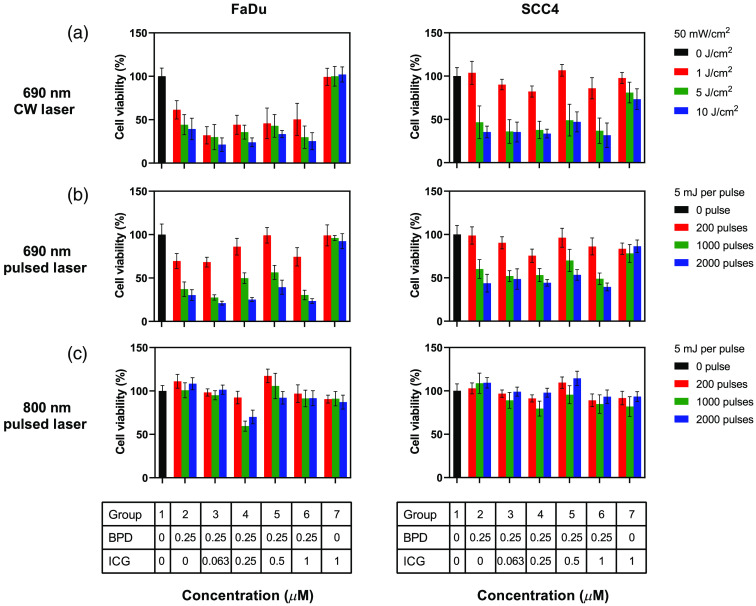
Efficacy of BPD PDT in the presence or absence of ICG in two cell lines (FaDu and SCC4). Response to PDT with (a) 690-nm CW laser, (b) 690-nm pulsed laser, and (c) 800-nm pulsed laser. PDT with 690 nm laser (CW or pulsed) gave a good treatment response compared to 800-nm pulsed laser. BPD’s PDT efficacy remained the same in the presence of varying concentrations of ICG. Error bars represent standard error of the mean (SEM) with a total of N=6 experimental repeats. One-way analysis of variance (ANOVA) Dunnett’s multiple comparison test was performed to identify statistically significant difference between the groups and p-value<0.01 was considered significant (Tables S1 and S2 in the Supplementary Material).

The viability of two different cell lines (FaDu and SCC4) evaluated 24 h post PDT with three different types of lasers and various ratios of the two dyes is shown in Fig. 3. The BPD concentration was kept constant at 0.25  μM except in the control “no-treatment” group (Fig. 3, group 1, black bars) and in the “no-BPD” group with 1  μM ICG concentration (Fig. 3, group 7). The ICG concentration was 0, 0.063, 0.25, 0.5, and 1  μM in groups 2 to 6, respectively. The results were normalized to the control no-treatment group 1 values. The 690 nm CW light irradiation at doses 1, 5, and $10\;J/{\mathrm{cm}}^{2}$ in group 7 showed 99.3±9.8%, 100±11.4%, and 102±8.8% of viable cells, respectively. The 690 and 800 nm pulsed irradiation showed cell viabilities to be 99±12.1%, 96±2.9%, 92.4±8.6%, 90.6±4.6%, 91.1±8.3%, and 87.1±8% for 200, 1000, and 2000 pulses, respectively. Overall, there was no statistically significant difference between the control group 1 and all the light doses in group 7 for the FaDu cells (Table S1 in the Supplementary Material) at different illumination wavelengths. On the other hand, SCC4 cells treated with ICG only [Fig. 3(a), right panel, group 7] showed very minimal response at higher light dose of $10\;J/{\mathrm{cm}}^{2}$ with 690 nm CW laser but had no statistically significant difference at a lower doses of 1 and $5\;J/{\mathrm{cm}}^{2}$, respectively (Table S2 in the Supplementary Material). We attribute this observation to ICG’s photosensitizing properties at higher light doses. For example, to achieve significant cell death, Abels et al.^33^ utilized longer incubation time (24 h), higher ICG concentration (>5  μM), and higher light dose at 800 nm ($80\;\mathrm{mW}/{\mathrm{cm}}^{2}$, $24\;J/{\mathrm{cm}}^{2}$) than the parameters used in our study (1  μM, 1 h accumulation, $50\;\mathrm{mW}/{\mathrm{cm}}^{2}$, $10\;J/{\mathrm{cm}}^{2}$). We also do not see ICG being phototoxic to both FaDu and SCC4 cells for pulsed irradiation at both wavelengths for all doses (Tables S1 and S2 in the Supplementary Material). The ICG concentration used in our study for PAI did not kill the cells, but it was high enough to give us a detectable PA signal.

### 3.2 Less Than 1 μM Indocyanine Green Concentration Had Minimal Impact on the Photodynamic Efficacy of Benzoporphyrin Derivative *In Vitro* for Both Pulsed and Continuous Wave Regimes

**Fig. 4:**
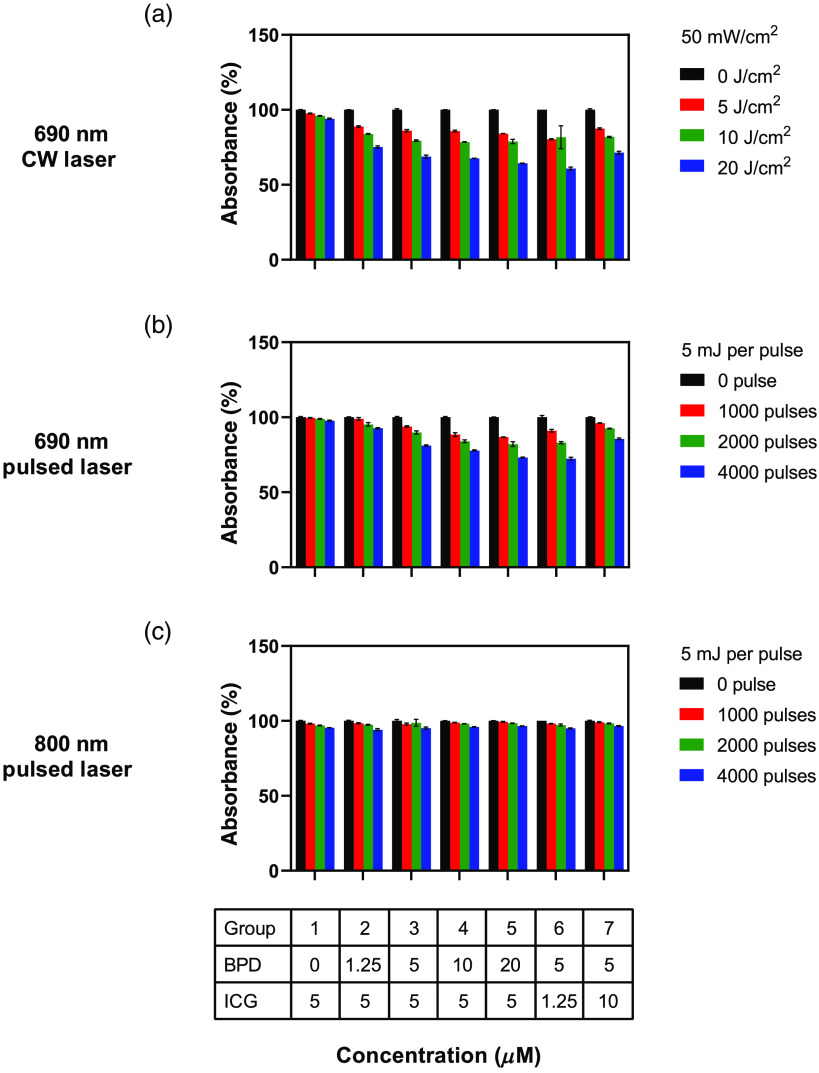
Bar graphs representing percentage decrease in absorbance of ICG at 780 nm. The solutions were irradiated with (a) CW laser at 690 nm and nanosecond pulsed laser at (b) 690 nm and (c) 800 nm, respectively. The error bars are SEM. Two-way ANOVA Tukey’s multiple comparison test was performed to identify statistically significant difference between the different conditions and p-value<0.05 was considered significant (see Table S3 for constant ICG concentration and Table S4 for constant BPD concentration).

To check if various ICG concentrations were influencing the PDT outcome at different illumination schemes, we compared group 1 (control, no-treatment, no dye) with groups 2 to 6. FaDu cells at 690 nm CW illumination at 1, 5, and $10\;J/{\mathrm{cm}}^{2}$ showed a significant difference between nontreated (group 1) and all treated groups (groups 2 to 6) containing BPD (Table S1 in the Supplementary Material). The same trend was observed for FaDu cells irradiated with 1000 and 2000 pulses at 690 nm. Low number of PA pulses (200 pulses at 690 nm pulsed irradiation) showed minimal or nonsignificant difference compared to the control group. SCC4 cells also had similar trends, except at $1\;J/{\mathrm{cm}}^{2}$ low-dose group CW 690 nm irradiation (Fig. 4, top-right panel, red bars). SCC4 and FaDu cells display different chemoresistance,^35^ and it is not surprising for these cells to display different PDT responses and the mechanisms involved are beyond the scope of this work and need to be studied further.

At high light doses, groups 2 to 6 showed no statistical difference between 690 nm CW or pulsed laser irradiation at both 690 and 800 nm (Tables S1 and S2 in the Supplementary Material). At lower light dose, pulsed irradiation caused less cell death than CW laser (Fig. 3, red bars, top and middle panel). These observations are in congruence with Pogue et al.^25^ and Kawauchi et al.^36^ Particularly for lower doses, the differences were observed, because pulsed PDT process consumes oxygen at a lower rate compared to CW laser, impacting singlet oxygen generation and therefore less therapeutic efficacy. Based on these observations and values, for the light doses and concentrations (below 1  μM) used, ICG did not influence BPD-PDT response.

### 3.3 Photoacoustic Monitoring of Indocyanine Green Photodegradation in the Presence of Benzoporphyrin Derivative

PA signal intensity is highly dependent on the optical absorption coefficient and concentration of the dye being imaged.^13^^,^^16^ It has been shown previously that ICG, a tricarbocyanine dye, decomposes with light (20 pulses of 10 ms duration and total energy of 50 J)^37^ and undergoes self-sensitized photodegradation. To evaluate if such an effect exists on ICG due to PA pulsed laser in the presence or absence of BPD, we performed experiments in solution and in tissue-mimicking phantoms.

The change in ICG absorbance 780 nm post irradiation with CW laser at 690 nm and nanosecond pulsed laser at 690 or 800 nm is shown in Fig. 4. Irradiation was performed on various solutions with varying concentrations of BPD (0 to 20  μM) and ICG (1.25 to 10  μM). BPD has low absorbance at 780 nm (Fig. 2) and hence the condition BPD: ICG 5: 0  μM is not included in these data. Irradiation at 800 nm caused minimal ICG photodegradation (less than 5% decrease in absorbance), irrespective of the BPD concentration and light dose [Fig. 4(c)]. A significant difference was observed only between the no-treatment group [Fig. 4(c), black bars] and highest light dose [Fig. 4(c), 4000 pulses, blue bars]. A greater degree of ICG photodegradation was observed for both CW and pulsed 690 nm irradiation, compared to 800 nm pulsed irradiation. The extent of photodegradation was maximum for higher light doses. In addition, at 690 nm irradiation, CW laser produced more ICG photodegradation than the pulsed laser. The ICG photodegradation increased with increase in BPD concentration in the solution (Fig. 4, groups 1 to 5). On the other hand, ICG photodegradation decreased with an increase in ICG concentration (Fig. 4, groups 3, 6, and 7). Similar trend (decrease in absorbance) was observed for ICG’s absorbance at 690 nm (Fig. S2 in the Supplementary Material), although the percentage decrease was lesser than 780 nm absorbance as expected.

**Fig. 5:**
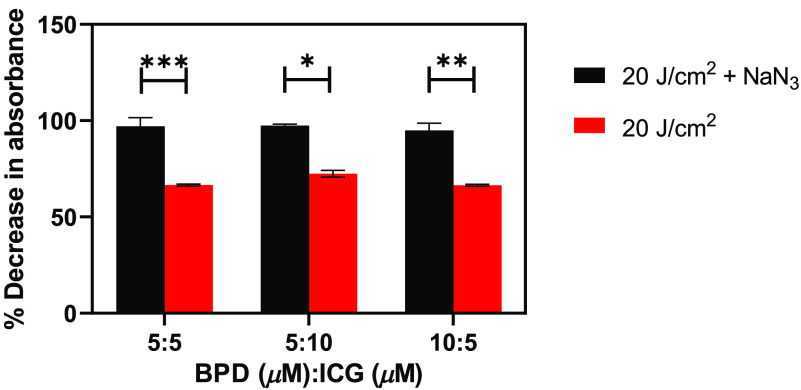
Bar graphs depicting percentage decrease in absorbance of the BPD and ICG mixture at varying concentrations in the presence (black bars) and absence (red bars) of ${\mathrm{NaN}}_{3}$ when treated with 690 nm CW laser. The error bars are SEM. Two-way ANOVA Tukey’s multiple comparison test was performed to identify statistically significant difference between the different conditions and p-value<0.05 was considered significant.

Next, we irradiated the mixture of BPD and ICG solutions in the presence and absence of sodium azide (${\mathrm{NaN}}_{3}$), a quencher of singlet oxygen, to reinforce our observations in Fig. 4. The percentage decrease in absorbance at 780 nm from three different BPD:ICG ratio solutions is demonstrated in Fig. 5. We observed ∼30% less decrease in absorbance in the presence of ${\mathrm{NaN}}_{3}$ (Fig. 5, black bars) than in the absence of ${\mathrm{NaN}}_{3}$ (Fig. 5, red bars). Irrespective of the BPD:ICG ratio, we observe that ${\mathrm{NaN}}_{3}$ attracts the BPD-generated singlet oxygen molecules before they can oxidize ICG.

**Fig. 6:**
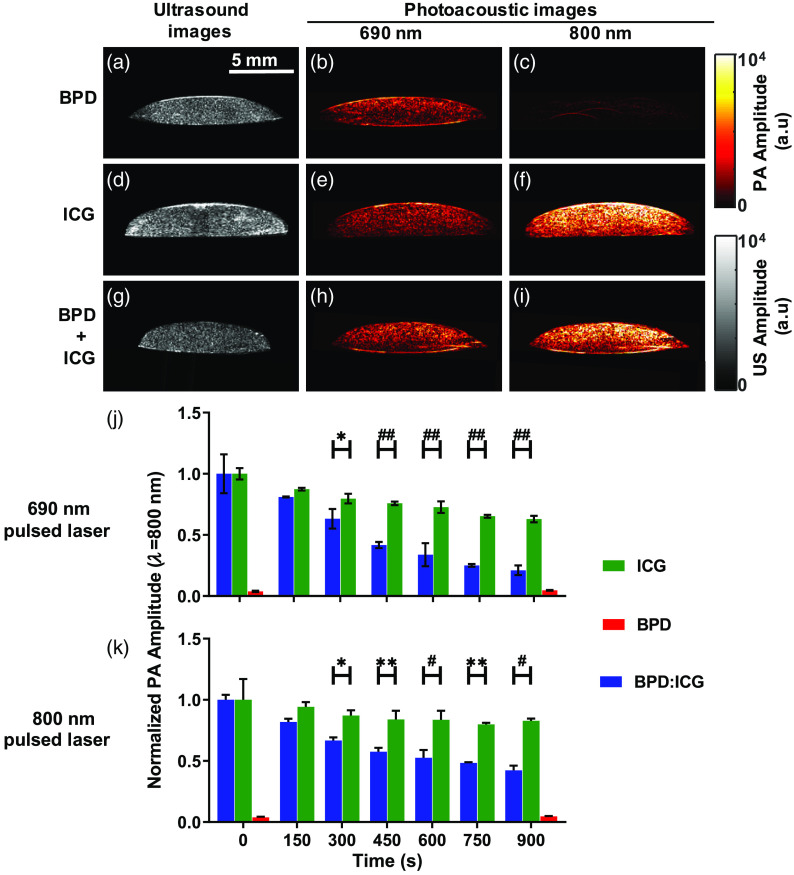
(a), (d), and (g) US and PA images at (b), (e), and (h) 690 nm and (c), (f), and (i) 800 nm of a tissue-mimicking phantom made of FaDu cells incubated with (a)–(c) 5  μM BPD (for 1 h), (d)–(f) 5  μM ICG (for 24 h), and (g)–(i) BPD and ICG combination for 1 and 24 h, respectively. (j) and (k) Normalized PA signal as a function of pulsed irradiation time (or PDT power), monitored at 800 nm; (j) irradiation at 690 nm for ICG 5  μM (green), BPD 5  μM (red), and BPD:ICG=5:5  μM (blue); (k) similar to (j) except with 800 nm irradiation. Error bars show the standard deviations from three separate measurements taken at each interval. BPD-only signal (red) was close to the noise level because of negligible absorption at 800 nm. Two-way ANOVA Sidak’s multiple comparison test was performed to identify statistically significant difference between the groups and *p*-value, where <0.05 was considered significant; * p<0.05, ** p<0.01, ^#^ p<0.001, and ^##^ p<0.0001.

Encouraged by the outcomes in Figs. 4 and 5, experiments were performed to determine if similar trends can be monitored with PAI in a tissue-mimicking environment. Phantoms containing FaDu cells incubated in either BPD, ICG, or a mixture (Fig. 6) underwent PAI at wavelengths 690, 800, and 900 nm, respectively. The PA images were plotted along with their US counterparts (Fig. 6, grayscale images, right panel) to facilitate comparison with the structural shape of the tissue-mimicking phantoms. As expected, the sample containing BPD alone had higher PA signal at 690 nm than at 800 nm [Figs. 6(b) and 6(c)]. The sample with ICG alone had higher PA signal at 800 nm than at 690 nm [Figs. 6(e) and 6(f)]. In agreement with absorbance curves shown in Fig. 2, the sample with both BPD and ICG had higher PA signal at 800 nm than at 690 nm [Figs. 6(h) and 6(i)] as molar absorption coefficient of ICG is greater than BPD.^38^^,^^39^ Next, we irradiated the sample with pulsed laser (690 or 800 nm) for ∼900  s to photoacoustically monitor ICG’s degradation. We observed the PA signal intensity decreases with pulsed laser exposure time as shown in Figs. 6(j) and 6(k). The presence of BPD [Figs. 6(j) and 6(k), blue bars] enhanced the ICG photodegradation in the mixture sample, i.e., the decrease in PA signal intensity was higher than in the sample with ICG alone [Figs. 6(j) and 6(k), green bars] and these differences were statistically significant. Furthermore, as expected from Figs. 4 and 5, the photodegradation with 690 nm pulsed irradiation is higher than 800 nm pulsed irradiation. These results clearly demonstrate that PAI can be used to monitor ICG photodegradation in the presence of singlet oxygen-generating molecules such as BPD.

BPD and ICG have molar extinction coefficient of $34,895\;M^{-1}\;{\mathrm{cm}}^{-1}$ at 687 nm in DMSO^39^ and $113790\;M^{-1}\;{\mathrm{cm}}^{-1}$ at 780 nm in water,^38^ respectively. Both the dyes have significantly higher absorption and can be distinguished from the endogenous chromophores such as hemoglobin via fluorescence^40^^,^^41^ or PAI.^13^^,^^42^^–^^45^ BPD is an excellent PS, whereas ICG needs very high PDT dose ($\mu M\times J/{\mathrm{cm}}^{2}$) due to its low singlet oxygen quantum yield. More than a regular PS behavior, ICG molecule relaxes via nonradiative mechanisms, such as heat, and hence is a very good PA or photothermal therapy agent.^22^ ICG is an interesting molecule with properties that heavily depend on the concentration and the surrounding environment.^38^^,^^46^ Several studies have shown that ICG demonstrates a blueshift in absorbance peak with an increase in the concentration or in the presence of serum, water, or salt, etc.^38^^,^^46^^–^^49^ and degrades when irradiated with light.^50^ Specifically, ICG undergoes conformational changes in the presence of reactive radicals that can either be self-generated or be generated by other molecules in the vicinity. J-aggregated ICG molecules or conjugation to plasmonic nanoparticles are shown to enhance ICG photostability.^51^^–^^53^ The photodegradation properties of ICG are also cleverly utilized for drug delivery strategies as reviewed recently by Gorka and Schnermann.^51^ Furthermore, ICG was shown to have enhanced photostability via encapsulation in lipids or association with plasmonic gold colloids.^54^^–^^56^ All these studies clearly point out the versatility of ICG as a theranostic agent and the need for thorough photostability characterization in the presence of other dyes or oxidizing agents in these formulations both in solutions and in cellular environment.

## 4 Conclusions

Here we evaluated the mutual impact of BPD and ICG on their PDT efficacy and PA signal stability. We observed no effect of ICG on the phototoxic capability of BPD at both 690- and 800-nm laser illumination. We also did not notice significant difference in BPD phototoxicity between CW and pulsed laser illumination at 690 nm in the presence of ICG. We, however, observed that the presence of BPD enhanced photodegradation of ICG, leading to a reduction in optical absorption and corresponding decrease in PA signal intensity at 800 nm. Thus far, we are the first group to study the interactions between BPD and ICG for both continuous and pulsed illumination. As BPD is primarily a type II PS that generates reactive oxygen species, it remains to be seen if ICG would react in a similar fashion in the presence of a type I PS that generates other reactive molecular species. Overall, the photointeractions between the two dyes in close proximity emphasizes the need to incorporate such studies prior to the design of multiagent nanoentities. Our future studies in tissue-mimicking phantoms and preclinical cancer models will involve incorporation of these dyes within nanoentities to shield one another from the oxidation effects observed here and further enhance the image contrast while preserving the PDT efficacy.

## References

[1] Triesscheijn M. et al., “Photodynamic therapy in oncology,” Oncologist 11, 1034–1044 (2006). 10.1634/theoncologist.11-9-1034 17030646

[r2] Agostinis P. et al., “Photodynamic therapy of cancer: an update,” CA Cancer J. Clin. 61, 250–281 (2011). CAMCAM 0007-9235 10.3322/caac.v61:4 21617154 PMC3209659

[r3] Dolmans D. E. Fukumura D. Jain R. K., “Photodynamic therapy for cancer,” Nat. Rev. Cancer 3, 380–387 (2003). NRCAC4 1474-175X 10.1038/nrc1071 12724736

[r4] Huang Z. et al., “Photodynamic therapy for treatment of solid tumors—potential and technical challenges,” Technol. Cancer Res. Treat. 7, 309–320 (2008). 10.1177/153303460800700405 18642969 PMC2593637

[5] Mallidi S. Spring B. Q. Hasan T., “Optical imaging, photodynamic therapy and optically triggered combination treatments,” Cancer J. 21, 194–205 (2015). 10.1097/PPO.0000000000000117 26049699 PMC4459538

[6] Huang Z., “A review of progress in clinical photodynamic therapy,” Technol. Cancer Res. Treat. 4, 283–293 (2005). 10.1177/153303460500400308 15896084 PMC1317568

[7] Celli J. P. et al., “Imaging and photodynamic therapy: mechanisms, monitoring, and optimization,” Chem. Rev. 110, 2795–2838 (2010). CHREAY 0009-2665 10.1021/cr900300p 20353192 PMC2896821

[8] Mallidi S. et al., “Platform for ergonomic intraoral photodynamic therapy using low-cost, modular 3D-printed components: design, comfort and clinical evaluation,” Sci. Rep. 9, 15830 (2019). SRCEC3 2045-2322 10.1038/s41598-019-51859-6 31676807 PMC6825190

[9] Pogue B. W. et al., “Revisiting photodynamic therapy dosimetry: reductionist & surrogate approaches to facilitate clinical success,” Phys. Med. Biol. 61, R57–R89 (2016). PHMBA7 0031-9155 10.1088/0031-9155/61/7/R57 26961864 PMC12153017

[10] Wilson B. C. Patterson M. S. Lilge L., “Implicit and explicit dosimetry in photodynamic therapy: a new paradigm,” Lasers Med. Sci. 12, 182–199 (1997). 10.1007/BF02765099 20803326

[11] Zhou X. et al., “Pretreatment photosensitizer dosimetry reduces variation in tumor response,” Int. J. Radiat. Oncol. Biol. Phys. 64, 1211–1220 (2006). IOBPD3 0360-3016 10.1016/j.ijrobp.2005.11.019 16504761

[12] Mallidi S. et al., “Photosensitizer fluorescence and singlet oxygen luminescence as dosimetric predictors of topical 5-aminolevulinic acid photodynamic therapy induced clinical erythema,” J. Biomed. Opt. 19, 028001 (2014). JBOPFO 1083-3668 10.1117/1.JBO.19.2.028001 24503639 PMC3915169

[13] Mallidi S. Luke G. P. Emelianov S., “Photoacoustic imaging in cancer detection, diagnosis, and treatment guidance,” Trends Biotechnol. 29, 213–221 (2011). TRBIDM 0167-7799 10.1016/j.tibtech.2011.01.006 21324541 PMC3080445

[r14] Xia J. Yao J. Wang L., “Photoacoustic tomography: principles and advances,” Electromagn. Waves 147, 1–22 (2014). 10.2528/PIER14032303 PMC4311576 25642127

[r15] Li M. Tang Y. Yao J., “Photoacoustic tomography of blood oxygenation: a mini review,” Photoacoustics 10, 65–73 (2018). 10.1016/j.pacs.2018.05.001 29988848 PMC6033062

[16] Xu M. Wang L. V., “Photoacoustic imaging in biomedicine,” Rev. Sci. Instrum. 77, 041101 (2006). RSINAK 0034-6748 10.1063/1.2195024

[17] Ho C. J. et al., “Multifunctional photosensitizer-based contrast agents for photoacoustic imaging,” Sci. Rep. 4, 5342 (2014). SRCEC3 2045-2322 10.1038/srep05342 24938638 PMC4061552

[18] Obaid G. et al., “Impacting pancreatic cancer therapy in heterotypic *in vitro* organoids and *in vivo* tumors with specificity-tuned, NIR-activable photoimmunonanoconjugates: towards conquering desmoplasia?” Nano Lett. 19, 7573–7587 (2019). NALEFD 1530-6984 10.1021/acs.nanolett.9b00859 31518145 PMC6934365

[19] Wang J. et al., “Switchable photoacoustic intensity of methylene blue via sodium dodecyl sulfate micellization,” Langmuir 34, 359–365 (2018). LANGD5 0743-7463 10.1021/acs.langmuir.7b03718 29232146 PMC6200325

[20] Wang J. et al., “A mechanistic investigation of methylene blue and heparin interactions and their photoacoustic enhancement,” Bioconjug. Chem. 29, 3768–3775 (2018). BCCHES 1043-1802 10.1021/acs.bioconjchem.8b00639 30281976 PMC8046596

[21] Wang H. et al., “Indocyanine green-incorporating nanoparticles for cancer theranostics,” Theranostics 8, 1227–1242 (2018). 10.7150/thno.22872 29507616 PMC5835932

[22] Giraudeau C. et al., “Indocyanine green: photosensitizer or chromophore? Still a debate,” Curr. Med. Chem. 21, 1871–1897 (2014). CMCHE7 0929-8673 10.2174/0929867321666131218095802 24350844

[23] Reindl S. et al., “Quantum yield of triplet formation for indocyanine green,” J. Photochem. Photobiol. A 105, 65–68 (1997). JPPCEJ 1010-6030 10.1016/S1010-6030(96)04584-4

[24] Tekrony A. D. et al., “Photobleaching kinetics of Verteporfin and Lemuteporfin in cells and optically trapped multilamellar vesicles using two-photon excitation,” Photochem. Photobiol. 87, 853–861 (2011). PHCBAP 0031-8655 10.1111/php.2011.87.issue-4 21488879

[25] Pogue B. W. et al., “Absorbed photodynamic dose from pulsed versus continuous wave light examined with tissue-simulating dosimeters,” Appl. Opt. 36, 7257–7214 (1997). APOPAI 0003-6935 10.1364/AO.36.007257 18264235

[26] Lin J. et al., “Photosensitizer-loaded gold vesicles with strong plasmonic coupling effect for imaging-guided photothermal/photodynamic therapy,” ACS Nano 7, 5320–5329 (2013). ANCAC3 1936-0851 10.1021/nn4011686 23721576 PMC3709863

[27] Xu H. et al., “PEGylated liposomal photosensitizers as theranostic agents for dual-modal photoacoustic and fluorescence imaging-guided photodynamic therapy,” J. Innov. Opt. Health Sci. 12(3), 1941003 (2019). 10.1142/S1793545819410037

[r28] Lemaster J. E. Jokerst J. V., “What is new in nanoparticle-based photoacoustic imaging?” WIREs Nanomed. Nanobiotechnol. 9 (2017). 10.1002/wnan.1404 PMC5045757 27038222

[r29] Zhang Y. Lovell J. F., “Recent applications of phthalocyanines and naphthalocyanines for imaging and therapy,” WIREs Nanomed. Nanobiotechnol. 9 (2016). 10.1002/wnan.1420 PMC5179311 27439671

[r30] Obaid G. et al., “Photonanomedicine: a convergence of photodynamic therapy and nanotechnology,” Nanoscale 8, 12471–12503 (2016). NANOHL 2040-3364 10.1039/C5NR08691D 27328309 PMC4956486

[31] Rai P. et al., “Development and applications of photo-triggered theranostic agents,” Adv. Drug Delivery Rev. 62, 1094–1124 (2010). ADDREP 0169-409X 10.1016/j.addr.2010.09.002 PMC2991599 20858520

[32] Huang H. C. et al., “Photodynamic therapy synergizes with irinotecan to overcome compensatory mechanisms and improve treatment outcomes in pancreatic cancer,” Cancer Res. 76, 1066–1077 (2016). CNREA8 0008-5472 10.1158/0008-5472.CAN-15-0391 26719532 PMC4775276

[r33] Abels C. et al., “Indocyanine green (ICG) and laser irradiation induce photooxidation,” Arch. Dermatol. Res. 292, 404–411 (2000). 10.1007/s004030000147 10994775

[34] Savellano M. D. Hasan T., “Photochemical targeting of epidermal growth factor receptor: a mechanistic study,” Clin. Cancer Res. 11, 1658–1668 (2005). 10.1158/1078-0432.CCR-04-1902 15746071

[35] Cullen K. J. et al., “Glutathione S-transferase π amplification is associated with cisplatin resistance in head and neck squamous cell carcinoma cell lines and primary tumors,” Cancer Res. 63, 8097–8102 (2003). CNREA8 0008-5472 14678959

[36] Kawauchi S. et al., “Differences between cytotoxicity in photodynamic therapy using a pulsed laser and a continuous wave laser: study of oxygen consumption and photobleaching,” Lasers Med. Sci. 18, 179–183 (2004). 10.1007/s10103-004-0288-8 15042420

[37] Engel E. et al., “Light-induced decomposition of indocyanine green,” Invest. Ophthalmol. Visual Sci. 49, 1777–1783 (2008). IOVSDA 0146-0404 10.1167/iovs.07-0911 18436812

[38] Landsman M. L. et al., “Light-absorbing properties, stability, and spectral stabilization of indocyanine green,” J. Appl. Physiol. 40, 575–583 (1976). 10.1152/jappl.1976.40.4.575 776922

[39] Aveline B. Hasan T. Redmond R. W., “Photophysical and photosensitizing properties of benzoporphyrin derivative monoacid ring A (BPD-MA),” Photochem. Photobiol. 59, 328–335 (1994). PHCBAP 0031-8655 10.1111/php.1994.59.issue-3 8016212

[40] Kraft J. C. Ho R. J. Y., “Interactions of indocyanine green and lipid in enhancing near-infrared fluorescence properties: the basis for near-infrared imaging *in vivo*,” Biochemistry 53, 1275–1283 (2014). 10.1021/bi500021j 24512123 PMC3985908

[41] Mordon S. et al., “Indocyanine green: physicochemical factors affecting its fluorescence *in vivo*,” Microvasc. Res. 55, 146–152 (1998). MIVRA6 0026-2862 10.1006/mvre.1998.2068 9521889

[42] Laramie M. D. et al., “Small molecule optoacoustic contrast agents: an unexplored avenue for enhancing in vivo imaging,” Molecules 23, 2766 (2018). 10.3390/molecules23112766 PMC6278390 30366395

[r43] Luke G. P. Yeager D. Emelianov S. Y., “Biomedical applications of photoacoustic imaging with exogenous contrast agents,” Ann. Biomed. Eng. 40, 422–437 (2012). ABMECF 0090-6964 10.1007/s10439-011-0449-4 22048668

[r44] Weber J. Beard P. C. Bohndiek S. E., “Contrast agents for molecular photoacoustic imaging,” Nat. Methods 13, 639–650 (2016).1548-7091 10.1038/nmeth.3929 27467727

[45] Fu Q. et al., “Photoacoustic imaging: contrast agents and their biomedical applications,” Adv. Mater. 31, 1805875 (2019). ADVMEW 0935-9648 10.1002/adma.201805875 30556205

[46] Holzer W. et al., “Photostability and thermal stability of indocyanine green,” J. Photochem. Photobiol. B 47, 155–164 (1998). JPPBEG 1011-1344 10.1016/S1011-1344(98)00216-4 10093915

[r47] Yuan B. H. Chen N. G. Zhu Q., “Emission and absorption properties of indocyanine green in Intralipid solution,” J. Biomed. Opt. 9, 497–503 (2004). JBOPFO 1083-3668 10.1117/1.1695411 15189087 PMC1533769

[r48] Mindt S. et al., “Stability and degradation of indocyanine green in plasma, aqueous solution and whole blood,” Photochem. Photobiol. Sci. 17, 1189–1196 (2018). PPSHCB 1474-905X 10.1039/C8PP00064F 30028469

[49] Kirchherr A. K. Briel A. Mader K., “Stabilization of indocyanine green by encapsulation within micellar systems,” Mol. Pharmaceutics 6, 480–491 (2009). MPOHBP 1543-8384 10.1021/mp8001649 19228053

[50] Engel E. et al., “Light-induced decomposition of indocyanine green,” Invest. Ophthalmol. Visual Sci. 49, 1777–1783 (2008). IOVSDA 0146-0404 10.1167/iovs.07-0911 18436812

[51] Gorka A. P. Schnermann M. J., “Harnessing cyanine photooxidation: from slowing photobleaching to near-IR uncaging,” Curr. Opin. Chem. Biol. 33, 117–125 (2016). COCBF4 1367-5931 10.1016/j.cbpa.2016.05.022 27348157 PMC7383357

[r52] Penha F. M. et al., “Biochemical analysis and decomposition products of indocyanine green in relation to solvents, dye concentrations and laser exposure,” Ophthalmologica 230, 59–67 (2013). OPHTAD 0030-3755 10.1159/000353871 24022720

[53] Chen J. et al., “Indocyanine green-loaded gold nanostars for sensitive SERS imaging and subcellular monitoring of photothermal therapy,” Nanoscale 9, 11888–11901 (2017). NANOHL 2040-3364 10.1039/C7NR02798B 28561825

[54] Geddes C. D. Cao H. S. Lakowicz J. R., “Enhanced photostability of ICG in close proximity to gold colloids,” Spectrochim. Acta, Part A 59, 2611–2617 (2003). SAMCAS 1386-1425 10.1016/S1386-1425(03)00015-5 PMC2753828 12963458

[r55] Yuan A. et al., “Activatable photodynamic destruction of cancer cells by NIR dye/photosensitizer loaded liposomes,” Chem. Commun. 51, 3340–3342 (2015). 10.1039/C4CC09689D 25619336

[56] Miranda D. et al., “Indocyanine green binds to DOTAP liposomes for enhanced optical properties and tumor photoablation,” Biomater. Sci. 7, 3158–3164 (2019). 10.1039/C9BM00551J 31232421 PMC6650340

