# Mutual impact of clinically translatable near-infrared dyes on photoacoustic image contrast and *in vitro* photodynamic therapy efficacy (Erratum)

**DOI:** 10.1117/1.JBO.25.6.069802

**Published:** 2020-06-30

**Authors:** Ljubica Z. Petrovic, Marvin Xavierselvan, Maju Kuriakose, Michael D. Kennedy, Christopher D. Nguyen, Julian J. Batt, Kelsey B. Detels, Srivalleesha Mallidi

**Affiliations:** Tufts University, Department of Biomedical Engineering, Medford, Massachusetts, United States

## Abstract

The erratum corrects an error in the *y*-axis labels of Fig. 4 and Fig. S2.

This article [*J. Biomed. Opt.* **25**(06), 0638008 (2020) doi: 10.1117/1.JBO.25.6.063808] was originally published online on 28 February 2020 with mislabeled *y*-axes in Fig. 4 and Fig. S2, panels (a)–(c). The correct label is “Absorbance (%).” The data in the figures remains the same.

This article was corrected online on 25 June 2020.

