# Supplementary material for: Mutual impact of clinically translatable near-infrared dyes on photoacoustic image contrast and in vitro photodynamic therapy efficacy
Source: J Biomed Opt. 2020 Feb 28;25(6):063808. doi: 10.1117/1.JBO.25.6.063808 (PMC7048201; doi:10.1117/1.JBO.25.6.063808)

Mutual impact of clinically translatable NIR dyes on photoacoustic image contrast

and *in vitro* photodynamic therapy efficacy

**Supplementary Figures**

**Fig. S1:** Photographs of the tissue mimicking phantoms made with gelatin base and cells only (A) or cells incubated with both BPD and ICG dyes (B).


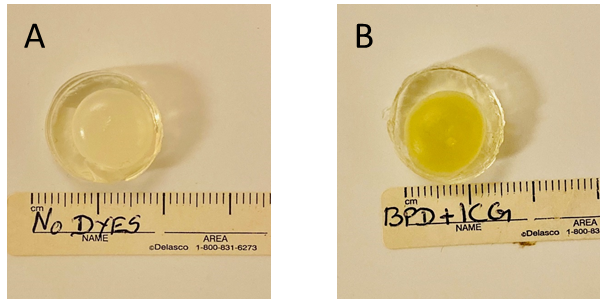


**Fig. S2:** Bar graphs representing percent decrease in absorbance of ICG at 690 nm in the presence or absence of BPD. In the left panel BPD concentration is varied with ICG concentration kept being constant at 5 µM and in right panel, ICG concentration is varied with BPD concentration kept constant at 5 µM. The solutions were irradiated with (A) continuous laser at 690 nm and nanosecond pulsed laser at B) 690 nm and C) 800 nm respectively.


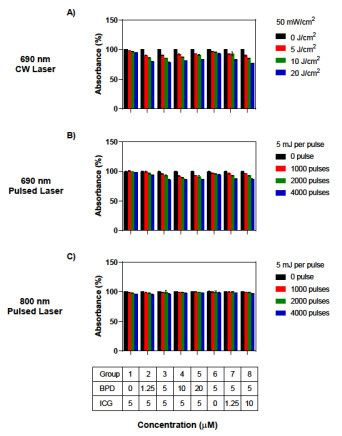

Supplement: Supplementary file 1 [file JBO_025_063808_SD001.docx]
