# Supplementary material for: Mutual impact of clinically translatable near-infrared dyes on photoacoustic image contrast and in vitro photodynamic therapy efficacy
Source: J Biomed Opt. 2020 Feb 28;25(6):063808. doi: 10.1117/1.JBO.25.6.063808 (PMC7048201; doi:10.1117/1.JBO.25.6.063808)
Supplement: Supplementary file 2 [file JBO_025_063808_SD002.docx]

Mutual impact of clinically translatable NIR dyes on photoacoustic image contrast and *in vitro* photodynamic therapy efficacy

**Supplementary Tables**

**Table S1:** Comparison of response in FaDu cells when PDT-treated with 690 nm CW laser, 690 nm and 800 nm pulsed laser using one-way ANOVA Dunnett’s multiple comparison test and p-value<0.01 was considered significant (Fig. 3).

| 690 nm  CW laser | BPD (µM): ICG (µM)  Group number | Mean Difference | Significant?  P < 0.01? | Summary | Adjusted P Value |
| --- | --- | --- | --- | --- | --- |
| 1 J/cm^2^ | NT vs 0.25:0  1 vs 2 | 0.1257 | Yes | ** | 0.0024 |
|  | NT vs 0.25:0.0625  1 vs 3 | 0.2443 | Yes | **** | <0.0001 |
|  | NT vs 0.25:0.25  1 vs 4 | 0.1953 | Yes | **** | <0.0001 |
|  | NT vs 0.25:0.5  1 vs 5 | 0.1887 | Yes | **** | <0.0001 |
|  | NT vs 0.25:1  1 vs 6 | 0.1703 | Yes | **** | <0.0001 |
|  | NT vs 0:1  1 vs 7 | -0.02767 | No | ns | 0.9042 |
|  | 0.25:0 vs 0.25:0.0625  2 vs 3 | 0.1187 | Yes | ** | 0.0094 |
|  | 0.25:0 vs 0.25:0.25  2 vs 4 | 0.06967 | No | ns | 0.1501 |
|  | 0.25:0 vs 0.25:0.5  2 vs 5 | 0.06300 | No | ns | 0.2118 |
|  | 0.25:0 vs 0.25:1  2 vs 6 | 0.04467 | No | ns | 0.4900 |
|  | 0.25:0 vs 0:1  2 vs 7 | -0.1533 | Yes | ** | 0.0014 |
|  | 0.25:0.0625 vs 0.25:0.25  3 vs 4 | -0.04900 | No | ns | 0.4048 |
|  | 0.25:0.0625 vs 0.25:0.5  3 vs 5 | -0.05567 | No | ns | 0.3064 |
|  | 0.25:0.0625 vs 0.25:1  3 vs 6 | -0.07400 | No | ns | 0.1308 |
|  | 0.25:0.0625 vs 0:1  3 vs 7 | -0.2720 | Yes | **** | <0.0001 |
|  | 0.25:0.25 vs 0.25:0.5  4 vs 5 | -0.006667 | No | ns | 0.9951 |
|  | 0.25:0.25 vs 0.25:1  4 vs 6 | -0.02500 | No | ns | 0.8265 |
|  | 0.25:0.25 vs 0:1  4 vs 7 | -0.2230 | Yes | *** | 0.0007 |
|  | 0.25:0.5 vs 0.25:1  5 vs 6 | -0.01833 | No | ns | 0.8622 |
|  | 0.25:0.5 vs 0:1  5 vs 7 | -0.2163 | Yes | ** | 0.0030 |
|  | 0.25:1 vs 0:1  6 vs 7 | -0.1980 | Yes | ** | 0.0046 |
| 5 J/cm^2^ | NT vs 0.25:0  1 vs 2 | 0.2303 | Yes | **** | <0.0001 |
|  | NT vs 0.25:0.0625  1 vs 3 | 0.2767 | Yes | **** | <0.0001 |
|  | NT vs 0.25:0.25  1 vs 4 | 0.2580 | Yes | **** | <0.0001 |
|  | NT vs 0.25:0.5  1 vs 5 | 0.2347 | Yes | **** | <0.0001 |
|  | NT vs 0.25:1  1 vs 6 | 0.2770 | Yes | **** | <0.0001 |
|  | NT vs 0:1  1 vs 7 | 0.04967 | No | ns | 0.3178 |
|  | 0.25:0 vs 0.25:0.0625  2 vs 3 | 0.04633 | No | ns | 0.1671 |
|  | 0.25:0 vs 0.25:0.25  2 vs 4 | 0.02767 | No | ns | 0.5766 |
|  | 0.25:0 vs 0.25:0.5  2 vs 5 | 0.004333 | No | ns | 0.9997 |
|  | 0.25:0 vs 0.25:1  2 vs 6 | 0.04667 | No | ns | 0.1629 |
|  | 0.25:0 vs 0:1  2 vs 7 | -0.1807 | Yes | **** | <0.0001 |
|  | 0.25:0.0625 vs 0.25:0.25  3 vs 4 | -0.01867 | No | ns | 0.8040 |
|  | 0.25:0.0625 vs 0.25:0.5  3 vs 5 | -0.04200 | No | ns | 0.2263 |
|  | 0.25:0.0625 vs 0.25:1  3 vs 6 | 0.0003333 | No | ns | >0.9999 |
|  | 0.25:0.0625 vs 0:1  3 vs 7 | -0.2270 | Yes | **** | <0.0001 |
|  | 0.25:0.25 vs 0.25:0.5  4 vs 5 | -0.02333 | No | ns | 0.6458 |
|  | 0.25:0.25 vs 0.25:1  4 vs 6 | 0.01900 | No | ns | 0.7611 |
|  | 0.25:0.25 vs 0:1  4 vs 7 | -0.2083 | Yes | **** | <0.0001 |
|  | 0.25:0.5 vs 0.25:1  5 vs 6 | 0.04233 | No | ns | 0.2706 |
|  | 0.25:0.5 vs 0:1  5 vs 7 | -0.1850 | Yes | *** | 0.0008 |
|  | 0.25:1 vs 0:1  6 vs 7 | -0.2273 | Yes | *** | 0.0003 |
| 10 J/cm^2^ | NT vs 0.25:0  1 vs 2 | 0.2193 | Yes | **** | <0.0001 |
|  | NT vs 0.25:0.0625  1 vs 3 | 0.2900 | Yes | **** | <0.0001 |
|  | NT vs 0.25:0.25  1 vs 4 | 0.2797 | Yes | **** | <0.0001 |
|  | NT vs 0.25:0.5  1 vs 5 | 0.2423 | Yes | **** | <0.0001 |
|  | NT vs 0.25:1  1 vs 6 | 0.2743 | Yes | **** | <0.0001 |
|  | NT vs 0:1  1 vs 7 | -0.02733 | No | ns | 0.8576 |
|  | 0.25:0 vs 0.25:0.0625  2 vs 3 | 0.07067 | No | * | 0.0311 |
|  | 0.25:0 vs 0.25:0.25  2 vs 4 | 0.06033 | No | ns | 0.0702 |
|  | 0.25:0 vs 0.25:0.5  2 vs 5 | 0.02300 | No | ns | 0.7548 |
|  | 0.25:0 vs 0.25:1  2 vs 6 | 0.05500 | No | ns | 0.1056 |
|  | 0.25:0 vs 0:1  2 vs 7 | -0.2467 | Yes | **** | <0.0001 |
|  | 0.25:0.0625 vs 0.25:0.25  3 vs 4 | -0.01033 | No | ns | 0.9663 |
|  | 0.25:0.0625 vs 0.25:0.5  3 vs 5 | -0.04767 | No | ns | 0.1475 |
|  | 0.25:0.0625 vs 0.25:1  3 vs 6 | -0.01567 | No | ns | 0.8749 |
|  | 0.25:0.0625 vs 0:1  3 vs 7 | -0.3173 | Yes | **** | <0.0001 |
|  | 0.25:0.25 vs 0.25:0.5  4 vs 5 | -0.03733 | No | ns | 0.3288 |
|  | 0.25:0.25 vs 0.25:1  4 vs 6 | -0.005333 | No | ns | 0.9915 |
|  | 0.25:0.25 vs 0:1  4 vs 7 | -0.3070 | Yes | **** | <0.0001 |
|  | 0.25:0.5 vs 0.25:1  5 vs 6 | 0.03200 | No | ns | 0.4423 |
|  | 0.25:0.5 vs 0:1  5 vs 7 | -0.2697 | Yes | *** | 0.0001 |
|  | 0.25:1 vs 0:1  6 vs 7 | -0.3017 | Yes | **** | <0.0001 |
|  |  |  |  |  |  |
| 690 nm pulsed laser  (no. of pulses) | BPD (µM): ICG (µM)  Group number | Mean Difference | Significant?  P < 0.01? | Summary | Adjusted P Value |
| 200 | NT vs 0.25:0  1 vs 2 | 0.1261 | Yes | ** | 0.0054 |
|  | NT vs 0.25:0.0625  1 vs 3 | 0.1304 | Yes | ** | 0.0040 |
|  | NT vs 0.25:0.25  1 vs 4 | 0.06944 | No | ns | 0.2159 |
|  | NT vs 0.25:0.5  1 vs 5 | 0.02444 | No | ns | 0.9621 |
|  | NT vs 0.25:1  1 vs 6 | 0.1094 | No | * | 0.0173 |
|  | NT vs 0:1  1 vs 7 | 0.02511 | No | ns | 0.9572 |
|  | 0.25:0 vs 0.25:0.0625  2 vs 3 | 0.004333 | No | ns | 0.9998 |
|  | 0.25:0 vs 0.25:0.25  2 vs 4 | -0.05667 | No | ns | 0.3136 |
|  | 0.25:0 vs 0.25:0.5  2 vs 5 | -0.1017 | No | * | 0.0299 |
|  | 0.25:0 vs 0.25:1  2 vs 6 | -0.01667 | No | ns | 0.9762 |
|  | 0.25:0 vs 0:1  2 vs 7 | -0.1010 | No | * | 0.0310 |
|  | 0.25:0.0625 vs 0.25:0.25  3 vs 4 | -0.06100 | No | ns | 0.2346 |
|  | 0.25:0.0625 vs 0.25:0.5  3 vs 5 | -0.1060 | No | * | 0.0248 |
|  | 0.25:0.0625 vs 0.25:1  3 vs 6 | -0.02100 | No | ns | 0.9073 |
|  | 0.25:0.0625 vs 0:1  3 vs 7 | -0.1053 | No | * | 0.0256 |
|  | 0.25:0.25 vs 0.25:0.5  4 vs 5 | -0.04500 | No | ns | 0.4846 |
|  | 0.25:0.25 vs 0.25:1  4 vs 6 | 0.04000 | No | ns | 0.5671 |
|  | 0.25:0.25 vs 0:1  4 vs 7 | -0.04433 | No | ns | 0.4952 |
|  | 0.25:0.5 vs 0.25:1  5 vs 6 | 0.08500 | No | ns | 0.1027 |
|  | 0.25:0.5 vs 0:1  5 vs 7 | 0.0006667 | No | ns | 0.9997 |
|  | 0.25:1 vs 0:1  6 vs 7 | -0.08433 | No | ns | 0.1194 |
| 1000 | NT vs 0.25:0  1 vs 2 | 0.2398 | Yes | **** | <0.0001 |
|  | NT vs 0.25:0.0625  1 vs 3 | 0.2718 | Yes | **** | <0.0001 |
|  | NT vs 0.25:0.25  1 vs 4 | 0.1971 | Yes | **** | <0.0001 |
|  | NT vs 0.25:0.5  1 vs 5 | 0.1751 | Yes | **** | <0.0001 |
|  | NT vs 0.25:1  1 vs 6 | 0.2621 | Yes | **** | <0.0001 |
|  | NT vs 0:1  1 vs 7 | 0.04211 | No | ns | 0.6560 |
|  | 0.25:0 vs 0.25:0.0625  2 vs 3 | 0.03200 | No | ns | 0.6692 |
|  | 0.25:0 vs 0.25:0.25  2 vs 4 | -0.04267 | No | ns | 0.4279 |
|  | 0.25:0 vs 0.25:0.5  2 vs 5 | -0.06467 | No | ns | 0.1276 |
|  | 0.25:0 vs 0.25:1  2 vs 6 | 0.02233 | No | ns | 0.8784 |
|  | 0.25:0 vs 0:1  2 vs 7 | -0.1977 | Yes | **** | <0.0001 |
|  | 0.25:0.0625 vs 0.25:0.25  3 vs 4 | -0.07467 | No | * | 0.0479 |
|  | 0.25:0.0625 vs 0.25:0.5  3 vs 5 | -0.09667 | No | * | 0.0119 |
|  | 0.25:0.0625 vs 0.25:1  3 vs 6 | -0.009667 | No | ns | 0.9856 |
|  | 0.25:0.0625 vs 0:1  3 vs 7 | -0.2297 | Yes | **** | <0.0001 |
|  | 0.25:0.25 vs 0.25:0.5  4 vs 5 | -0.02200 | No | ns | 0.7838 |
|  | 0.25:0.25 vs 0.25:1  4 vs 6 | 0.06500 | No | ns | 0.1206 |
|  | 0.25:0.25 vs 0:1  4 vs 7 | -0.1550 | Yes | ** | 0.0016 |
|  | 0.25:0.5 vs 0.25:1  5 vs 6 | 0.08700 | No | ns | 0.0559 |
|  | 0.25:0.5 vs 0:1  5 vs 7 | -0.1330 | Yes | ** | 0.0097 |
|  | 0.25:1 vs 0:1  6 vs 7 | -0.2200 | Yes | *** | 0.0008 |
| 2000 | NT vs 0.25:0  1 vs 2 | 0.2384 | Yes | **** | <0.0001 |
|  | NT vs 0.25:0.0625  1 vs 3 | 0.2771 | Yes | **** | <0.0001 |
|  | NT vs 0.25:0.25  1 vs 4 | 0.2598 | Yes | **** | <0.0001 |
|  | NT vs 0.25:0.5  1 vs 5 | 0.2008 | Yes | **** | <0.0001 |
|  | NT vs 0.25:1  1 vs 6 | 0.2668 | Yes | **** | <0.0001 |
|  | NT vs 0:1  1 vs 7 | -0.01922 | No | ns | 0.9831 |
|  | 0.25:0 vs 0.25:0.0625  2 vs 3 | 0.03867 | No | ns | 0.4587 |
|  | 0.25:0 vs 0.25:0.25  2 vs 4 | 0.02133 | No | ns | 0.8715 |
|  | 0.25:0 vs 0.25:0.5  2 vs 5 | -0.03767 | No | ns | 0.4816 |
|  | 0.25:0 vs 0.25:1  2 vs 6 | 0.02833 | No | ns | 0.7125 |
|  | 0.25:0 vs 0:1  2 vs 7 | -0.2577 | Yes | **** | <0.0001 |
|  | 0.25:0.0625 vs 0.25:0.25  3 vs 4 | -0.01733 | No | ns | 0.8778 |
|  | 0.25:0.0625 vs 0.25:0.5  3 vs 5 | -0.07633 | No | * | 0.0310 |
|  | 0.25:0.0625 vs 0.25:1  3 vs 6 | -0.01033 | No | ns | 0.9769 |
|  | 0.25:0.0625 vs 0:1  3 vs 7 | -0.2963 | Yes | **** | <0.0001 |
|  | 0.25:0.25 vs 0.25:0.5  4 vs 5 | -0.05900 | No | ns | 0.1303 |
|  | 0.25:0.25 vs 0.25:1  4 vs 6 | 0.007000 | No | ns | 0.9864 |
|  | 0.25:0.25 vs 0:1  4 vs 7 | -0.2790 | Yes | **** | <0.0001 |
|  | 0.25:0.5 vs 0.25:1  5 vs 6 | 0.06600 | No | ns | 0.1223 |
|  | 0.25:0.5 vs 0:1  5 vs 7 | -0.2200 | Yes | *** | 0.0006 |
|  | 0.25:1 vs 0:1  6 vs 7 | -0.2860 | Yes | *** | 0.0002 |
|  |  |  |  |  |  |
| 800 nm pulsed laser  (no. of pulses) | BPD (µM): ICG (µM)  Group number | Mean Difference | Significant?  P < 0.01? | Summary | Adjusted P Value |
| 200 | NT vs 0.25:0  1 vs 2 | -0.03889 | No | ns | 0.4712 |
|  | NT vs 0.25:0.0625  1 vs 3 | 0.005778 | No | ns | 0.9997 |
|  | NT vs 0.25:0.25  1 vs 4 | -0.06056 | No | ns | 0.0977 |
|  | NT vs 0.25:0.5  1 vs 5 | 0.02611 | No | ns | 0.8217 |
|  | NT vs 0.25:1  1 vs 6 | 0.01044 | No | ns | 0.9974 |
|  | NT vs 0:1  1 vs 7 | 0.03244 | No | ns | 0.6529 |
|  | 0.25:0 vs 0.25:0.0625  2 vs 3 | 0.04467 | No | ns | 0.5408 |
|  | 0.25:0 vs 0.25:0.25  2 vs 4 | -0.02167 | No | ns | 0.9406 |
|  | 0.25:0 vs 0.25:0.5  2 vs 5 | 0.06500 | No | ns | 0.2309 |
|  | 0.25:0 vs 0.25:1  2 vs 6 | 0.04933 | No | ns | 0.4546 |
|  | 0.25:0 vs 0:1  2 vs 7 | 0.07133 | No | ns | 0.1702 |
|  | 0.25:0.0625 vs 0.25:0.25  3 vs 4 | -0.06633 | No | ns | 0.1727 |
|  | 0.25:0.0625 vs 0.25:0.5  3 vs 5 | 0.02033 | No | ns | 0.9113 |
|  | 0.25:0.0625 vs 0.25:1  3 vs 6 | 0.004667 | No | ns | 0.9997 |
|  | 0.25:0.0625 vs 0:1  3 vs 7 | 0.02667 | No | ns | 0.8098 |
|  | 0.25:0.25 vs 0.25:0.5  4 vs 5 | 0.08667 | No | ns | 0.0867 |
|  | 0.25:0.25 vs 0.25:1  4 vs 6 | 0.07100 | No | ns | 0.1688 |
|  | 0.25:0.25 vs 0:1  4 vs 7 | 0.09300 | No | ns | 0.0660 |
|  | 0.25:0.5 vs 0.25:1  5 vs 6 | -0.01567 | No | ns | 0.8468 |
|  | 0.25:0.5 vs 0:1  5 vs 7 | 0.006333 | No | ns | 0.9721 |
|  | 0.25:1 vs 0:1  6 vs 7 | 0.02200 | No | ns | 0.7301 |
| 1000 | NT vs 0.25:0  1 vs 2 | 0.01544 | No | ns | 0.9872 |
|  | NT vs 0.25:0.0625  1 vs 3 | 0.03444 | No | ns | 0.6711 |
|  | NT vs 0.25:0.25  1 vs 4 | -0.0008889 | No | ns | >0.9999 |
|  | NT vs 0.25:0.5  1 vs 5 | 0.1514 | Yes | **** | <0.0001 |
|  | NT vs 0.25:1  1 vs 6 | 0.04644 | No | ns | 0.3704 |
|  | NT vs 0:1  1 vs 7 | 0.04744 | No | ns | 0.3489 |
|  | 0.25:0 vs 0.25:0.0625  2 vs 3 | 0.01900 | No | ns | 0.9762 |
|  | 0.25:0 vs 0.25:0.25  2 vs 4 | -0.01633 | No | ns | 0.9874 |
|  | 0.25:0 vs 0.25:0.5  2 vs 5 | 0.1360 | No | * | 0.0112 |
|  | 0.25:0 vs 0.25:1  2 vs 6 | 0.03100 | No | ns | 0.8578 |
|  | 0.25:0 vs 0:1  2 vs 7 | 0.03200 | No | ns | 0.8432 |
|  | 0.25:0.0625 vs 0.25:0.25  3 vs 4 | -0.03533 | No | ns | 0.7436 |
|  | 0.25:0.0625 vs 0.25:0.5  3 vs 5 | 0.1170 | No | * | 0.0312 |
|  | 0.25:0.0625 vs 0.25:1  3 vs 6 | 0.01200 | No | ns | 0.9916 |
|  | 0.25:0.0625 vs 0:1  3 vs 7 | 0.01300 | No | ns | 0.9886 |
|  | 0.25:0.25 vs 0.25:0.5  4 vs 5 | 0.1523 | No | * | 0.0147 |
|  | 0.25:0.25 vs 0.25:1  4 vs 6 | 0.04733 | No | ns | 0.5466 |
|  | 0.25:0.25 vs 0:1  4 vs 7 | 0.04833 | No | ns | 0.5321 |
|  | 0.25:0.5 vs 0.25:1  5 vs 6 | -0.1050 | No | * | 0.0136 |
|  | 0.25:0.5 vs 0:1  5 vs 7 | -0.1040 | No | * | 0.0142 |
|  | 0.25:1 vs 0:1  6 vs 7 | 0.001000 | No | ns | 0.9990 |
| 2000 | NT vs 0.25:0  1 vs 2 | -0.04856 | No | ns | 0.1334 |
|  | NT vs 0.25:0.0625  1 vs 3 | -0.02322 | No | ns | 0.7960 |
|  | NT vs 0.25:0.25  1 vs 4 | 0.01078 | No | ns | 0.9932 |
|  | NT vs 0.25:0.5  1 vs 5 | 0.09144 | Yes | ** | 0.0013 |
|  | NT vs 0.25:1  1 vs 6 | 0.01244 | No | ns | 0.9856 |
|  | NT vs 0:1  1 vs 7 | 0.02911 | No | ns | 0.6062 |
|  | 0.25:0 vs 0.25:0.0625  2 vs 3 | 0.02533 | No | ns | 0.7969 |
|  | 0.25:0 vs 0.25:0.25  2 vs 4 | 0.05933 | No | ns | 0.1502 |
|  | 0.25:0 vs 0.25:0.5  2 vs 5 | 0.1400 | Yes | *** | 0.0008 |
|  | 0.25:0 vs 0.25:1  2 vs 6 | 0.06100 | No | ns | 0.1354 |
|  | 0.25:0 vs 0:1  2 vs 7 | 0.07767 | No | * | 0.0457 |
|  | 0.25:0.0625 vs 0.25:0.25  3 vs 4 | 0.03400 | No | ns | 0.5212 |
|  | 0.25:0.0625 vs 0.25:0.5  3 vs 5 | 0.1147 | Yes | ** | 0.0043 |
|  | 0.25:0.0625 vs 0.25:1  3 vs 6 | 0.03567 | No | ns | 0.4832 |
|  | 0.25:0.0625 vs 0:1  3 vs 7 | 0.05233 | No | ns | 0.1993 |
|  | 0.25:0.25 vs 0.25:0.5  4 vs 5 | 0.08067 | No | ns | 0.0520 |
|  | 0.25:0.25 vs 0.25:1  4 vs 6 | 0.001667 | No | ns | 0.9999 |
|  | 0.25:0.25 vs 0:1  4 vs 7 | 0.01833 | No | ns | 0.8541 |
|  | 0.25:0.5 vs 0.25:1  5 vs 6 | -0.07900 | No | ns | 0.0596 |
|  | 0.25:0.5 vs 0:1  5 vs 7 | -0.06233 | No | ns | 0.1272 |
|  | 0.25:1 vs 0:1  6 vs 7 | 0.01667 | No | ns | 0.7966 |
|  |  |  |  |  |  |

**Table S2:** Comparison of response of SCC4 cells to PDT with 690 nm CW laser, 690 nm and 800 nm pulsed laser using one-way ANOVA Dunnett’s multiple comparison test (Fig 3)

| 690 nm  CW laser | BPD (µM): ICG (µM)  Group number | Mean Difference | Significant?  P < 0.01? | Summary | Adjusted P Value |
| --- | --- | --- | --- | --- | --- |
| 1 J/cm^2^ | NT vs 0.25:0  1 vs 2 | -0.01600 | No | ns | 0.9524 |
|  | NT vs 0.25:0.0625  1 vs 3 | 0.02100 | No | ns | 0.8560 |
|  | NT vs 0.25:0.25  1 vs 4 | 0.04300 | No | ns | 0.2203 |
|  | NT vs 0.25:0.5  1 vs 5 | -0.02400 | No | ns | 0.7720 |
|  | NT vs 0.25:1  1 vs 6 | 0.03267 | No | ns | 0.4880 |
|  | NT vs 0:1  1 vs 7 | 0.0003333 | No | ns | >0.9999 |
|  | 0.25:0 vs 0.25:0.0625  2 vs 3 | 0.03700 | No | ns | 0.4962 |
|  | 0.25:0 vs 0.25:0.25  2 vs 4 | 0.05900 | No | ns | 0.1419 |
|  | 0.25:0 vs 0.25:0.5  2 vs 5 | -0.008000 | No | ns | 0.9973 |
|  | 0.25:0 vs 0.25:1  2 vs 6 | 0.04867 | No | ns | 0.2669 |
|  | 0.25:0 vs 0:1  2 vs 7 | 0.01633 | No | ns | 0.9493 |
|  | 0.25:0.0625 vs 0.25:0.25  3 vs 4 | 0.02200 | No | ns | 0.5991 |
|  | 0.25:0.0625 vs 0.25:0.5  3 vs 5 | -0.04500 | No | ns | 0.1056 |
|  | 0.25:0.0625 vs 0.25:1  3 vs 6 | 0.01167 | No | ns | 0.9188 |
|  | 0.25:0.0625 vs 0:1  3 vs 7 | -0.02067 | No | ns | 0.6450 |
|  | 0.25:0.25 vs 0.25:0.5  4 vs 5 | -0.06700 | No | * | 0.0279 |
|  | 0.25:0.25 vs 0.25:1  4 vs 6 | -0.01033 | No | ns | 0.9207 |
|  | 0.25:0.25 vs 0:1  4 vs 7 | -0.04267 | No | ns | 0.1613 |
|  | 0.25:0.5 vs 0.25:1  5 vs 6 | 0.05667 | No | ns | 0.0846 |
|  | 0.25:0.5 vs 0:1  5 vs 7 | 0.02433 | No | ns | 0.5067 |
|  | 0.25:1 vs 0:1  6 vs 7 | -0.03233 | No | ns | 0.3369 |
| 5 J/cm^2^ | NT vs 0.25:0  1 vs 2 | 0.1417 | Yes | **** | <0.0001 |
|  | NT vs 0.25:0.0625  1 vs 3 | 0.1703 | Yes | **** | <0.0001 |
|  | NT vs 0.25:0.25  1 vs 4 | 0.1657 | Yes | **** | <0.0001 |
|  | NT vs 0.25:0.5  1 vs 5 | 0.1353 | Yes | **** | <0.0001 |
|  | NT vs 0.25:1  1 vs 6 | 0.1680 | Yes | **** | <0.0001 |
|  | NT vs 0:1  1 vs 7 | 0.04967 | No | ns | 0.0572 |
|  | 0.25:0 vs 0.25:0.0625  2 vs 3 | 0.02867 | No | ns | 0.5146 |
|  | 0.25:0 vs 0.25:0.25  2 vs 4 | 0.02400 | No | ns | 0.6613 |
|  | 0.25:0 vs 0.25:0.5  2 vs 5 | -0.006333 | No | ns | 0.9973 |
|  | 0.25:0 vs 0.25:1  2 vs 6 | 0.02633 | No | ns | 0.5869 |
|  | 0.25:0 vs 0:1  2 vs 7 | -0.09200 | Yes | ** | 0.0030 |
|  | 0.25:0.0625 vs 0.25:0.25  3 vs 4 | -0.004667 | No | ns | 0.9967 |
|  | 0.25:0.0625 vs 0.25:0.5  3 vs 5 | -0.03500 | No | ns | 0.2347 |
|  | 0.25:0.0625 vs 0.25:1  3 vs 6 | -0.002333 | No | ns | 0.9998 |
|  | 0.25:0.0625 vs 0:1  3 vs 7 | -0.1207 | Yes | *** | 0.0002 |
|  | 0.25:0.25 vs 0.25:0.5  4 vs 5 | -0.03033 | No | ns | 0.3345 |
|  | 0.25:0.25 vs 0.25:1  4 vs 6 | 0.002333 | No | ns | 0.9986 |
|  | 0.25:0.25 vs 0:1  4 vs 7 | -0.1160 | Yes | *** | 0.0009 |
|  | 0.25:0.5 vs 0.25:1  5 vs 6 | 0.03267 | No | ns | 0.3135 |
|  | 0.25:0.5 vs 0:1  5 vs 7 | -0.08567 | No | * | 0.0154 |
|  | 0.25:1 vs 0:1  6 vs 7 | -0.1183 | Yes | ** | 0.0033 |
| 10 J/cm^2^ | NT vs 0.25:0  1 vs 2 | 0.1750 | Yes | **** | <0.0001 |
|  | NT vs 0.25:0.0625  1 vs 3 | 0.1750 | Yes | **** | <0.0001 |
|  | NT vs 0.25:0.25  1 vs 4 | 0.1797 | Yes | **** | <0.0001 |
|  | NT vs 0.25:0.5  1 vs 5 | 0.1443 | Yes | **** | <0.0001 |
|  | NT vs 0.25:1  1 vs 6 | 0.1847 | Yes | **** | <0.0001 |
|  | NT vs 0:1  1 vs 7 | 0.07633 | Yes | *** | 0.0008 |
|  | 0.25:0 vs 0.25:0.0625  2 vs 3 | 0.000 | No | ns | >0.9999 |
|  | 0.25:0 vs 0.25:0.25  2 vs 4 | 0.004667 | No | ns | 0.9985 |
|  | 0.25:0 vs 0.25:0.5  2 vs 5 | -0.03067 | No | ns | 0.3133 |
|  | 0.25:0 vs 0.25:1  2 vs 6 | 0.009667 | No | ns | 0.9684 |
|  | 0.25:0 vs 0:1  2 vs 7 | -0.09867 | Yes | *** | 0.0004 |
|  | 0.25:0.0625 vs 0.25:0.25  3 vs 4 | 0.004667 | No | ns | 0.9969 |
|  | 0.25:0.0625 vs 0.25:0.5  3 vs 5 | -0.03067 | No | ns | 0.3407 |
|  | 0.25:0.0625 vs 0.25:1  3 vs 6 | 0.009667 | No | ns | 0.9561 |
|  | 0.25:0.0625 vs 0:1  3 vs 7 | -0.09867 | Yes | ** | 0.0012 |
|  | 0.25:0.25 vs 0.25:0.5  4 vs 5 | -0.03533 | No | ns | 0.2419 |
|  | 0.25:0.25 vs 0.25:1  4 vs 6 | 0.005000 | No | ns | 0.9878 |
|  | 0.25:0.25 vs 0:1  4 vs 7 | -0.1033 | Yes | ** | 0.0020 |
|  | 0.25:0.5 vs 0.25:1  5 vs 6 | 0.04033 | No | ns | 0.2051 |
|  | 0.25:0.5 vs 0:1  5 vs 7 | -0.06800 | No | * | 0.0410 |
|  | 0.25:1 vs 0:1  6 vs 7 | -0.1083 | Yes | ** | 0.0053 |
|  |  |  |  |  |  |
| 690 nm pulsed laser  (no. of pulses) | BPD (µM): ICG (µM)  Group number | Mean Difference | Significant?  P < 0.01? | Summary | Adjusted P Value |
| 200 | NT vs 0.25:0  1 vs 2 | -0.02822 | No | ns | 0.7774 |
|  | NT vs 0.25:0.0625  1 vs 3 | -0.003222 | No | ns | 0.9999 |
|  | NT vs 0.25:0.25  1 vs 4 | 0.04078 | No | ns | 0.4336 |
|  | NT vs 0.25:0.5  1 vs 5 | -0.02056 | No | ns | 0.9323 |
|  | NT vs 0.25:1  1 vs 6 | 0.009111 | No | ns | 0.9983 |
|  | NT vs 0:1  1 vs 7 | 0.01711 | No | ns | 0.9702 |
|  | 0.25:0 vs 0.25:0.0625  2 vs 3 | 0.02500 | No | ns | 0.7697 |
|  | 0.25:0 vs 0.25:0.25  2 vs 4 | 0.06900 | No | ns | 0.0616 |
|  | 0.25:0 vs 0.25:0.5  2 vs 5 | 0.007667 | No | ns | 0.9974 |
|  | 0.25:0 vs 0.25:1  2 vs 6 | 0.03733 | No | ns | 0.4556 |
|  | 0.25:0 vs 0:1  2 vs 7 | 0.04533 | No | ns | 0.2925 |
|  | 0.25:0.0625 vs 0.25:0.25  3 vs 4 | 0.04400 | No | ns | 0.2637 |
|  | 0.25:0.0625 vs 0.25:0.5  3 vs 5 | -0.01733 | No | ns | 0.8785 |
|  | 0.25:0.0625 vs 0.25:1  3 vs 6 | 0.01233 | No | ns | 0.9579 |
|  | 0.25:0.0625 vs 0:1  3 vs 7 | 0.02033 | No | ns | 0.8122 |
|  | 0.25:0.25 vs 0.25:0.5  4 vs 5 | -0.06133 | No | ns | 0.1151 |
|  | 0.25:0.25 vs 0.25:1  4 vs 6 | -0.03167 | No | ns | 0.5237 |
|  | 0.25:0.25 vs 0:1  4 vs 7 | -0.02367 | No | ns | 0.7102 |
|  | 0.25:0.5 vs 0.25:1  5 vs 6 | 0.02967 | No | ns | 0.4990 |
|  | 0.25:0.5 vs 0:1  5 vs 7 | 0.03767 | No | ns | 0.3560 |
|  | 0.25:1 vs 0:1  6 vs 7 | 0.008000 | No | ns | 0.9417 |
| 1000 | NT vs 0.25:0  1 vs 2 | 0.1218 | Yes | *** | 0.0001 |
|  | NT vs 0.25:0.0625  1 vs 3 | 0.1411 | Yes | **** | <0.0001 |
|  | NT vs 0.25:0.25  1 vs 4 | 0.1384 | Yes | **** | <0.0001 |
|  | NT vs 0.25:0.5  1 vs 5 | 0.09844 | Yes | ** | 0.0015 |
|  | NT vs 0.25:1  1 vs 6 | 0.1484 | Yes | **** | <0.0001 |
|  | NT vs 0:1  1 vs 7 | 0.07944 | No | * | 0.0108 |
|  | 0.25:0 vs 0.25:0.0625  2 vs 3 | 0.01933 | No | ns | 0.8034 |
|  | 0.25:0 vs 0.25:0.25  2 vs 4 | 0.01667 | No | ns | 0.8747 |
|  | 0.25:0 vs 0.25:0.5  2 vs 5 | -0.02333 | No | ns | 0.6798 |
|  | 0.25:0 vs 0.25:1  2 vs 6 | 0.02667 | No | ns | 0.5731 |
|  | 0.25:0 vs 0:1  2 vs 7 | -0.04233 | No | ns | 0.1977 |
|  | 0.25:0.0625 vs 0.25:0.25  3 vs 4 | -0.002667 | No | ns | 0.9997 |
|  | 0.25:0.0625 vs 0.25:0.5  3 vs 5 | -0.04267 | No | ns | 0.1264 |
|  | 0.25:0.0625 vs 0.25:1  3 vs 6 | 0.007333 | No | ns | 0.9825 |
|  | 0.25:0.0625 vs 0:1  3 vs 7 | -0.06167 | No | * | 0.0237 |
|  | 0.25:0.25 vs 0.25:0.5  4 vs 5 | -0.04000 | No | ns | 0.1934 |
|  | 0.25:0.25 vs 0.25:1  4 vs 6 | 0.01000 | No | ns | 0.9266 |
|  | 0.25:0.25 vs 0:1  4 vs 7 | -0.05900 | No | * | 0.0491 |
|  | 0.25:0.5 vs 0.25:1  5 vs 6 | 0.05000 | No | ns | 0.1104 |
|  | 0.25:0.5 vs 0:1  5 vs 7 | -0.01900 | No | ns | 0.6229 |
|  | 0.25:1 vs 0:1  6 vs 7 | -0.06900 | No | * | 0.0363 |
| 2000 | NT vs 0.25:0  1 vs 2 | 0.1501 | Yes | **** | <0.0001 |
|  | NT vs 0.25:0.0625  1 vs 3 | 0.1374 | Yes | **** | <0.0001 |
|  | NT vs 0.25:0.25  1 vs 4 | 0.1488 | Yes | **** | <0.0001 |
|  | NT vs 0.25:0.5  1 vs 5 | 0.1251 | Yes | *** | 0.0003 |
|  | NT vs 0.25:1  1 vs 6 | 0.1611 | Yes | **** | <0.0001 |
|  | NT vs 0:1  1 vs 7 | 0.03911 | No | ns | 0.4753 |
|  | 0.25:0 vs 0.25:0.0625  2 vs 3 | -0.01267 | No | ns | 0.9781 |
|  | 0.25:0 vs 0.25:0.25  2 vs 4 | -0.001333 | No | ns | >0.9999 |
|  | 0.25:0 vs 0.25:0.5  2 vs 5 | -0.02500 | No | ns | 0.7672 |
|  | 0.25:0 vs 0.25:1  2 vs 6 | 0.01100 | No | ns | 0.9880 |
|  | 0.25:0 vs 0:1  2 vs 7 | -0.1110 | Yes | ** | 0.0031 |
|  | 0.25:0.0625 vs 0.25:0.25  3 vs 4 | 0.01133 | No | ns | 0.9559 |
|  | 0.25:0.0625 vs 0.25:0.5  3 vs 5 | -0.01233 | No | ns | 0.9419 |
|  | 0.25:0.0625 vs 0.25:1  3 vs 6 | 0.02367 | No | ns | 0.6635 |
|  | 0.25:0.0625 vs 0:1  3 vs 7 | -0.09833 | Yes | ** | 0.0038 |
|  | 0.25:0.25 vs 0.25:0.5  4 vs 5 | -0.02367 | No | ns | 0.2297 |
|  | 0.25:0.25 vs 0.25:1  4 vs 6 | 0.01233 | No | ns | 0.6699 |
|  | 0.25:0.25 vs 0:1  4 vs 7 | -0.1097 | Yes | **** | <0.0001 |
|  | 0.25:0.5 vs 0.25:1  5 vs 6 | 0.03600 | No | ns | 0.0772 |
|  | 0.25:0.5 vs 0:1  5 vs 7 | -0.08600 | Yes | ** | 0.0017 |
|  | 0.25:1 vs 0:1  6 vs 7 | -0.1220 | Yes | *** | 0.0003 |
|  |  |  |  |  |  |
| 800 nm pulsed laser  (no. of pulses) | BPD (µM): ICG (µM)  Group number | Mean Difference | Significant?  P < 0.01? | Summary | Adjusted P Value |
| 200 | NT vs 0.25:0  1 vs 2 | -0.03689 | No | ns | 0.4915 |
|  | NT vs 0.25:0.0625  1 vs 3 | -0.01522 | No | ns | 0.9789 |
|  | NT vs 0.25:0.25  1 vs 4 | -0.06056 | No | ns | 0.0822 |
|  | NT vs 0.25:0.5  1 vs 5 | 0.004444 | No | ns | 0.9997 |
|  | NT vs 0.25:1  1 vs 6 | 0.01244 | No | ns | 0.9926 |
|  | NT vs 0:1  1 vs 7 | 0.003111 | No | ns | 0.9999 |
|  | 0.25:0 vs 0.25:0.0625  2 vs 3 | 0.02167 | No | ns | 0.8404 |
|  | 0.25:0 vs 0.25:0.25  2 vs 4 | -0.02367 | No | ns | 0.7936 |
|  | 0.25:0 vs 0.25:0.5  2 vs 5 | 0.04133 | No | ns | 0.3553 |
|  | 0.25:0 vs 0.25:1  2 vs 6 | 0.04933 | No | ns | 0.2191 |
|  | 0.25:0 vs 0:1  2 vs 7 | 0.04000 | No | ns | 0.3828 |
|  | 0.25:0.0625 vs 0.25:0.25  3 vs 4 | -0.04533 | No | ns | 0.2437 |
|  | 0.25:0.0625 vs 0.25:0.5  3 vs 5 | 0.01967 | No | ns | 0.8281 |
|  | 0.25:0.0625 vs 0.25:1  3 vs 6 | 0.02767 | No | ns | 0.6225 |
|  | 0.25:0.0625 vs 0:1  3 vs 7 | 0.01833 | No | ns | 0.8578 |
|  | 0.25:0.25 vs 0.25:0.5  4 vs 5 | 0.06500 | No | ns | 0.0929 |
|  | 0.25:0.25 vs 0.25:1  4 vs 6 | 0.07300 | No | ns | 0.0592 |
|  | 0.25:0.25 vs 0:1  4 vs 7 | 0.06367 | No | ns | 0.1001 |
|  | 0.25:0.5 vs 0.25:1  5 vs 6 | 0.008000 | No | ns | 0.9289 |
|  | 0.25:0.5 vs 0:1  5 vs 7 | -0.001333 | No | ns | 0.9979 |
|  | 0.25:1 vs 0:1  6 vs 7 | -0.009333 | No | ns | 0.9051 |
| 1000 | NT vs 0.25:0  1 vs 2 | 0.01211 | No | ns | 0.9927 |
|  | NT vs 0.25:0.0625  1 vs 3 | 0.06978 | No | * | 0.0298 |
|  | NT vs 0.25:0.25  1 vs 4 | 0.05078 | No | ns | 0.1690 |
|  | NT vs 0.25:0.5  1 vs 5 | 0.09778 | Yes | ** | 0.0018 |
|  | NT vs 0.25:1  1 vs 6 | 0.08244 | Yes | ** | 0.0084 |
|  | NT vs 0:1  1 vs 7 | 0.09078 | Yes | ** | 0.0036 |
|  | 0.25:0 vs 0.25:0.0625  2 vs 3 | 0.05767 | No | ns | 0.1000 |
|  | 0.25:0 vs 0.25:0.25  2 vs 4 | 0.03867 | No | ns | 0.3628 |
|  | 0.25:0 vs 0.25:0.5  2 vs 5 | 0.08567 | No | * | 0.0119 |
|  | 0.25:0 vs 0.25:1  2 vs 6 | 0.07033 | No | * | 0.0385 |
|  | 0.25:0 vs 0:1  2 vs 7 | 0.07867 | No | * | 0.0203 |
|  | 0.25:0.0625 vs 0.25:0.25  3 vs 4 | -0.01900 | No | ns | 0.7326 |
|  | 0.25:0.0625 vs 0.25:0.5  3 vs 5 | 0.02800 | No | ns | 0.4467 |
|  | 0.25:0.0625 vs 0.25:1  3 vs 6 | 0.01267 | No | ns | 0.9098 |
|  | 0.25:0.0625 vs 0:1  3 vs 7 | 0.02100 | No | ns | 0.6672 |
|  | 0.25:0.25 vs 0.25:0.5  4 vs 5 | 0.04700 | No | ns | 0.1271 |
|  | 0.25:0.25 vs 0.25:1  4 vs 6 | 0.03167 | No | ns | 0.3555 |
|  | 0.25:0.25 vs 0:1  4 vs 7 | 0.04000 | No | ns | 0.2065 |
|  | 0.25:0.5 vs 0.25:1  5 vs 6 | -0.01533 | No | ns | 0.6782 |
|  | 0.25:0.5 vs 0:1  5 vs 7 | -0.007000 | No | ns | 0.9157 |
|  | 0.25:1 vs 0:1  6 vs 7 | 0.008333 | No | ns | 0.8838 |
| 2000 | NT vs 0.25:0  1 vs 2 | -0.04322 | No | ns | 0.3064 |
|  | NT vs 0.25:0.0625  1 vs 3 | -0.008222 | No | ns | 0.9984 |
|  | NT vs 0.25:0.25  1 vs 4 | -0.06056 | No | ns | 0.0724 |
|  | NT vs 0.25:0.5  1 vs 5 | -0.004222 | No | ns | 0.9998 |
|  | NT vs 0.25:1  1 vs 6 | 0.01178 | No | ns | 0.9936 |
|  | NT vs 0:1  1 vs 7 | 0.01111 | No | ns | 0.9950 |
|  | 0.25:0 vs 0.25:0.0625  2 vs 3 | 0.03500 | No | ns | 0.4524 |
|  | 0.25:0 vs 0.25:0.25  2 vs 4 | -0.01733 | No | ns | 0.9079 |
|  | 0.25:0 vs 0.25:0.5  2 vs 5 | 0.03900 | No | ns | 0.3594 |
|  | 0.25:0 vs 0.25:1  2 vs 6 | 0.05500 | No | ns | 0.1237 |
|  | 0.25:0 vs 0:1  2 vs 7 | 0.05433 | No | ns | 0.1297 |
|  | 0.25:0.0625 vs 0.25:0.25  3 vs 4 | -0.05233 | No | ns | 0.1479 |
|  | 0.25:0.0625 vs 0.25:0.5  3 vs 5 | 0.004000 | No | ns | 0.9991 |
|  | 0.25:0.0625 vs 0.25:1  3 vs 6 | 0.02000 | No | ns | 0.8118 |
|  | 0.25:0.0625 vs 0:1  3 vs 7 | 0.01933 | No | ns | 0.8278 |
|  | 0.25:0.25 vs 0.25:0.5  4 vs 5 | 0.05633 | No | ns | 0.1391 |
|  | 0.25:0.25 vs 0.25:1  4 vs 6 | 0.07233 | No | ns | 0.0554 |
|  | 0.25:0.25 vs 0:1  4 vs 7 | 0.07167 | No | ns | 0.0576 |
|  | 0.25:0.5 vs 0.25:1  5 vs 6 | 0.01600 | No | ns | 0.6571 |
|  | 0.25:0.5 vs 0:1  5 vs 7 | 0.01533 | No | ns | 0.6778 |
|  | 0.25:1 vs 0:1  6 vs 7 | -0.0006667 | No | ns | 0.9993 |

**Table S3:** Comparison of ICG absorbance at 780 nm among samples treated with different doses of PDT with 690 nm CW laser, 690 nm and 800 nm pulsed laser using two-way ANOVA Tukey’s multiple comparison test and p-value<0.05 was considered significant (Fig 4).

| BPD (µM): ICG (µM) | 690 nm  CW laser | Mean Difference | Summary | Adjusted  p-value |
| --- | --- | --- | --- | --- |
| 0:5 | 0 vs 5 J/cm^2^ | 0.03033 | ns | 0.0699 |
|  | 0 vs 10 J/cm^2^ | 0.04900 | *** | 0.0009 |
|  | 0 vs 20 J/cm^2^ | 0.07367 | **** | <0.0001 |
|  | 5 vs 10 J/cm^2^ | 0.01867 | ns | 0.4198 |
|  | 5 vs 20 J/cm^2^ | 0.04333 | ** | 0.0039 |
|  | 10 vs 20 J/cm^2^ | 0.02467 | ns | 0.1866 |
| 1.25:5 | 0 vs 5 J/cm^2^ | 0.1400 | **** | <0.0001 |
|  | 0 vs 10 J/cm^2^ | 0.2010 | **** | <0.0001 |
|  | 0 vs 20 J/cm^2^ | 0.3090 | **** | <0.0001 |
|  | 5 vs 10 J/cm^2^ | 0.06100 | **** | <0.0001 |
|  | 5 vs 20 J/cm^2^ | 0.1690 | **** | <0.0001 |
|  | 10 vs 20 J/cm^2^ | 0.1080 | **** | <0.0001 |
| 5:5 | 0 vs 5 J/cm^2^ | 0.1680 | **** | <0.0001 |
|  | 0 vs 10 J/cm^2^ | 0.2467 | **** | <0.0001 |
|  | 0 vs 20 J/cm^2^ | 0.3750 | **** | <0.0001 |
|  | 5 vs 10 J/cm^2^ | 0.07867 | **** | <0.0001 |
|  | 5 vs 20 J/cm^2^ | 0.2070 | **** | <0.0001 |
|  | 10 vs 20 J/cm^2^ | 0.1283 | **** | <0.0001 |
| 10:5 | 0 vs 5 J/cm^2^ | 0.1720 | **** | <0.0001 |
|  | 0 vs 10 J/cm^2^ | 0.2617 | **** | <0.0001 |
|  | 0 vs 20 J/cm^2^ | 0.3940 | **** | <0.0001 |
|  | 5 vs 10 J/cm^2^ | 0.08967 | **** | <0.0001 |
|  | 5 vs 20 J/cm^2^ | 0.2220 | **** | <0.0001 |
|  | 10 vs 20 J/cm^2^ | 0.1323 | **** | <0.0001 |
| 20:5 | 0 vs 5 J/cm^2^ | 0.1907 | **** | <0.0001 |
|  | 0 vs 10 J/cm^2^ | 0.2537 | **** | <0.0001 |
|  | 0 vs 20 J/cm^2^ | 0.4280 | **** | <0.0001 |
|  | 5 vs 10 J/cm^2^ | 0.06300 | **** | <0.0001 |
|  | 5 vs 20 J/cm^2^ | 0.2373 | **** | <0.0001 |
|  | 10 vs 20 J/cm^2^ | 0.1743 | **** | <0.0001 |
| 5:1.25 | 0 vs 5 J/cm^2^ | 0.06433 | **** | <0.0001 |
|  | 0 vs 10 J/cm^2^ | 0.06000 | **** | <0.0001 |
|  | 0 vs 20 J/cm^2^ | 0.1280 | **** | <0.0001 |
|  | 5 vs 10 J/cm^2^ | -0.004333 | ns | 0.9841 |
|  | 5 vs 20 J/cm^2^ | 0.06367 | **** | <0.0001 |
|  | 10 vs 20 J/cm^2^ | 0.06800 | **** | <0.0001 |
| 5:10 | 0 vs 5 J/cm^2^ | 0.2847 | **** | <0.0001 |
|  | 0 vs 10 J/cm^2^ | 0.4087 | **** | <0.0001 |
|  | 0 vs 20 J/cm^2^ | 0.6450 | **** | <0.0001 |
|  | 5 vs 10 J/cm^2^ | 0.1240 | **** | <0.0001 |
|  | 5 vs 20 J/cm^2^ | 0.3603 | **** | <0.0001 |
|  | 10 vs 20 J/cm^2^ | 0.2363 | **** | <0.0001 |
|  |  |  |  |  |
| BPD (µM): ICG (µM) | 690 nm  pulsed laser | Mean Difference | Summary | Adjusted  p-value |
| 0:5 | 0 vs 1000 | 0.006333 | ns | 0.9550 |
|  | 0 vs 2000 | 0.01633 | ns | 0.5481 |
|  | 0 vs 4000 | 0.03367 | * | 0.0398 |
|  | 1000 vs 2000 | 0.01000 | ns | 0.8475 |
|  | 1000 vs 4000 | 0.02733 | ns | 0.1288 |
|  | 2000 vs 4000 | 0.01733 | ns | 0.4977 |
| 1.25:5 | 0 vs 1000 | 0.01533 | ns | 0.5990 |
|  | 0 vs 2000 | 0.07033 | **** | <0.0001 |
|  | 0 vs 4000 | 0.1087 | **** | <0.0001 |
|  | 1000 vs 2000 | 0.05500 | *** | 0.0002 |
|  | 1000 vs 4000 | 0.09333 | **** | <0.0001 |
|  | 2000 vs 4000 | 0.03833 | * | 0.0147 |
| 5:5 | 0 vs 1000 | 0.08867 | **** | <0.0001 |
|  | 0 vs 2000 | 0.1460 | **** | <0.0001 |
|  | 0 vs 4000 | 0.2730 | **** | <0.0001 |
|  | 1000 vs 2000 | 0.05733 | *** | 0.0001 |
|  | 1000 vs 4000 | 0.1843 | **** | <0.0001 |
|  | 2000 vs 4000 | 0.1270 | **** | <0.0001 |
| 10:5 | 0 vs 1000 | 0.1657 | **** | <0.0001 |
|  | 0 vs 2000 | 0.2297 | **** | <0.0001 |
|  | 0 vs 4000 | 0.3173 | **** | <0.0001 |
|  | 1000 vs 2000 | 0.06400 | **** | <0.0001 |
|  | 1000 vs 4000 | 0.1517 | **** | <0.0001 |
|  | 2000 vs 4000 | 0.08767 | **** | <0.0001 |
| 20:5 | 0 vs 1000 | 0.1887 | **** | <0.0001 |
|  | 0 vs 2000 | 0.2553 | **** | <0.0001 |
|  | 0 vs 4000 | 0.3833 | **** | <0.0001 |
|  | 1000 vs 2000 | 0.06667 | **** | <0.0001 |
|  | 1000 vs 4000 | 0.1947 | **** | <0.0001 |
|  | 2000 vs 4000 | 0.1280 | **** | <0.0001 |
| 5:1.25 | 0 vs 1000 | 0.03533 | * | 0.0282 |
|  | 0 vs 2000 | 0.06700 | **** | <0.0001 |
|  | 0 vs 4000 | 0.1097 | **** | <0.0001 |
|  | 1000 vs 2000 | 0.03167 | ns | 0.0590 |
|  | 1000 vs 4000 | 0.07433 | **** | <0.0001 |
|  | 2000 vs 4000 | 0.04267 | ** | 0.0054 |
| 5:10 | 0 vs 1000 | 0.1077 | **** | <0.0001 |
|  | 0 vs 2000 | 0.2063 | **** | <0.0001 |
|  | 0 vs 4000 | 0.3977 | **** | <0.0001 |
|  | 1000 vs 2000 | 0.09867 | **** | <0.0001 |
|  | 1000 vs 4000 | 0.2900 | **** | <0.0001 |
|  | 2000 vs 4000 | 0.1913 | **** | <0.0001 |
|  |  |  |  |  |
| BPD (µM): ICG (µM) | 800 nm  pulsed laser | Mean Difference | Summary | Adjusted  p-value |
| 0:5 | 0 vs 1000 | 0.02233 | ns | 0.0534 |
|  | 0 vs 2000 | 0.03767 | *** | 0.0003 |
|  | 0 vs 4000 | 0.05600 | **** | <0.0001 |
|  | 1000 vs 2000 | 0.01533 | ns | 0.2847 |
|  | 1000 vs 4000 | 0.03367 | ** | 0.0012 |
|  | 2000 vs 4000 | 0.01833 | ns | 0.1499 |
| 1.25:5 | 0 vs 1000 | 0.01967 | ns | 0.1086 |
|  | 0 vs 2000 | 0.03267 | ** | 0.0018 |
|  | 0 vs 4000 | 0.07367 | **** | <0.0001 |
|  | 1000 vs 2000 | 0.01300 | ns | 0.4296 |
|  | 1000 vs 4000 | 0.05400 | **** | <0.0001 |
|  | 2000 vs 4000 | 0.04100 | **** | <0.0001 |
| 5:5 | 0 vs 1000 | 0.02567 | * | 0.0198 |
|  | 0 vs 2000 | 0.01567 | ns | 0.2667 |
|  | 0 vs 4000 | 0.05767 | **** | <0.0001 |
|  | 1000 vs 2000 | -0.01000 | ns | 0.6461 |
|  | 1000 vs 4000 | 0.03200 | ** | 0.0023 |
|  | 2000 vs 4000 | 0.04200 | **** | <0.0001 |
| 10:5 | 0 vs 1000 | 0.01367 | ns | 0.3851 |
|  | 0 vs 2000 | 0.02333 | * | 0.0401 |
|  | 0 vs 4000 | 0.05000 | **** | <0.0001 |
|  | 1000 vs 2000 | 0.009667 | ns | 0.6702 |
|  | 1000 vs 4000 | 0.03633 | *** | 0.0004 |
|  | 2000 vs 4000 | 0.02667 | * | 0.0144 |
| 20:5 | 0 vs 1000 | 0.007667 | ns | 0.8051 |
|  | 0 vs 2000 | 0.01867 | ns | 0.1386 |
|  | 0 vs 4000 | 0.04167 | **** | <0.0001 |
|  | 1000 vs 2000 | 0.01100 | ns | 0.5727 |
|  | 1000 vs 4000 | 0.03400 | ** | 0.0011 |
|  | 2000 vs 4000 | 0.02300 | * | 0.0442 |
| 5:1.25 | 0 vs 1000 | 0.006000 | ns | 0.8951 |
|  | 0 vs 2000 | 0.009000 | ns | 0.7174 |
|  | 0 vs 4000 | 0.01633 | ns | 0.2330 |
|  | 1000 vs 2000 | 0.003000 | ns | 0.9849 |
|  | 1000 vs 4000 | 0.01033 | ns | 0.6217 |
|  | 2000 vs 4000 | 0.007333 | ns | 0.8251 |
| 5:10 | 0 vs 1000 | 0.02000 | ns | 0.0998 |
|  | 0 vs 2000 | 0.03700 | *** | 0.0003 |
|  | 0 vs 4000 | 0.07733 | **** | <0.0001 |
|  | 1000 vs 2000 | 0.01700 | ns | 0.2023 |
|  | 1000 vs 4000 | 0.05733 | **** | <0.0001 |
|  | 2000 vs 4000 | 0.04033 | **** | <0.0001 |

**Table. S4:** Comparison of ICG absorbance at 690 nm among samples treated with different doses of PDT with 690 nm CW laser, 690 nm and 800 nm pulsed laser using two-way ANOVA Tukey’s multiple comparison test (Fig S2)

| BPD (µM): ICG (µM) | 690 nm  CW laser | Mean Difference | Summary | Adjusted  p-value |
| --- | --- | --- | --- | --- |
| 0:5 | 0 vs 5 J/cm^2^ | 0.005667 | ns | 0.3776 |
|  | 0 vs 10 J/cm^2^ | 0.009667 | * | 0.0374 |
|  | 0 vs 20 J/cm^2^ | 0.01567 | *** | 0.0002 |
|  | 5 vs 10 J/cm^2^ | 0.004000 | ns | 0.6664 |
|  | 5 vs 20 J/cm^2^ | 0.01000 | * | 0.0293 |
|  | 10 vs 20 J/cm^2^ | 0.006000 | ns | 0.3271 |
| 1.25:5 | 0 vs 5 J/cm^2^ | 0.03233 | **** | <0.0001 |
|  | 0 vs 10 J/cm^2^ | 0.04567 | **** | <0.0001 |
|  | 0 vs 20 J/cm^2^ | 0.06767 | **** | <0.0001 |
|  | 5 vs 10 J/cm^2^ | 0.01333 | ** | 0.0018 |
|  | 5 vs 20 J/cm^2^ | 0.03533 | **** | <0.0001 |
|  | 10 vs 20 J/cm^2^ | 0.02200 | **** | <0.0001 |
| 5:5 | 0 vs 5 J/cm^2^ | 0.03667 | **** | <0.0001 |
|  | 0 vs 10 J/cm^2^ | 0.05533 | **** | <0.0001 |
|  | 0 vs 20 J/cm^2^ | 0.08500 | **** | <0.0001 |
|  | 5 vs 10 J/cm^2^ | 0.01867 | **** | <0.0001 |
|  | 5 vs 20 J/cm^2^ | 0.04833 | **** | <0.0001 |
|  | 10 vs 20 J/cm^2^ | 0.02967 | **** | <0.0001 |
| 10:5 | 0 vs 5 J/cm^2^ | 0.03767 | **** | <0.0001 |
|  | 0 vs 10 J/cm^2^ | 0.06067 | **** | <0.0001 |
|  | 0 vs 20 J/cm^2^ | 0.09067 | **** | <0.0001 |
|  | 5 vs 10 J/cm^2^ | 0.02300 | **** | <0.0001 |
|  | 5 vs 20 J/cm^2^ | 0.05300 | **** | <0.0001 |
|  | 10 vs 20 J/cm^2^ | 0.03000 | **** | <0.0001 |
| 20:5 | 0 vs 5 J/cm^2^ | 0.04567 | **** | <0.0001 |
|  | 0 vs 10 J/cm^2^ | 0.05733 | **** | <0.0001 |
|  | 0 vs 20 J/cm^2^ | 0.1050 | **** | <0.0001 |
|  | 5 vs 10 J/cm^2^ | 0.01167 | ** | 0.0078 |
|  | 5 vs 20 J/cm^2^ | 0.05933 | **** | <0.0001 |
|  | 10 vs 20 J/cm^2^ | 0.04767 | **** | <0.0001 |
| 5:0 | 0 vs 5 J/cm^2^ | 0.004333 | ns | 0.6072 |
|  | 0 vs 10 J/cm^2^ | 0.005667 | ns | 0.3776 |
|  | 0 vs 20 J/cm^2^ | 0.009000 | ns | 0.0596 |
|  | 5 vs 10 J/cm^2^ | 0.001333 | ns | 0.9812 |
|  | 5 vs 20 J/cm^2^ | 0.004667 | ns | 0.5475 |
|  | 10 vs 20 J/cm^2^ | 0.003333 | ns | 0.7780 |
| 5:1.25 | 0 vs 5 J/cm^2^ | 0.01400 | *** | 0.0010 |
|  | 0 vs 10 J/cm^2^ | 0.01333 | ** | 0.0018 |
|  | 0 vs 20 J/cm^2^ | 0.03167 | **** | <0.0001 |
|  | 5 vs 10 J/cm^2^ | -0.0006667 | ns | 0.9975 |
|  | 5 vs 20 J/cm^2^ | 0.01767 | **** | <0.0001 |
|  | 10 vs 20 J/cm^2^ | 0.01833 | **** | <0.0001 |
| 5:10 | 0 vs 5 J/cm^2^ | 0.06300 | **** | <0.0001 |
|  | 0 vs 10 J/cm^2^ | 0.09133 | **** | <0.0001 |
|  | 0 vs 20 J/cm^2^ | 0.1457 | **** | <0.0001 |
|  | 5 vs 10 J/cm^2^ | 0.02833 | **** | <0.0001 |
|  | 5 vs 20 J/cm^2^ | 0.08267 | **** | <0.0001 |
|  | 10 vs 20 J/cm^2^ | 0.05433 | **** | <0.0001 |
|  |  |  |  |  |
| BPD (µM): ICG (µM) | 690 nm  pulsed laser | Mean Difference | Summary | Adjusted  p-value |
| 0:5 | 0 vs 1000 | -0.002333 | ns | 0.9451 |
|  | 0 vs 2000 | 0.002000 | ns | 0.9644 |
|  | 0 vs 4000 | 0.005667 | ns | 0.5380 |
|  | 1000 vs 2000 | 0.004333 | ns | 0.7333 |
|  | 1000 vs 4000 | 0.008000 | ns | 0.2386 |
|  | 2000 vs 4000 | 0.003667 | ns | 0.8200 |
| 1.25:5 | 0 vs 1000 | 0.001333 | ns | 0.9889 |
|  | 0 vs 2000 | 0.01467 | ** | 0.0049 |
|  | 0 vs 4000 | 0.02333 | **** | <0.0001 |
|  | 1000 vs 2000 | 0.01333 | * | 0.0124 |
|  | 1000 vs 4000 | 0.02200 | **** | <0.0001 |
|  | 2000 vs 4000 | 0.008667 | ns | 0.1783 |
| 5:5 | 0 vs 1000 | 0.01833 | *** | 0.0003 |
|  | 0 vs 2000 | 0.02867 | **** | <0.0001 |
|  | 0 vs 4000 | 0.05933 | **** | <0.0001 |
|  | 1000 vs 2000 | 0.01033 | ns | 0.0774 |
|  | 1000 vs 4000 | 0.04100 | **** | <0.0001 |
|  | 2000 vs 4000 | 0.03067 | **** | <0.0001 |
| 10:5 | 0 vs 1000 | 0.03967 | **** | <0.0001 |
|  | 0 vs 2000 | 0.05367 | **** | <0.0001 |
|  | 0 vs 4000 | 0.07100 | **** | <0.0001 |
|  | 1000 vs 2000 | 0.01400 | ** | 0.0078 |
|  | 1000 vs 4000 | 0.03133 | **** | <0.0001 |
|  | 2000 vs 4000 | 0.01733 | *** | 0.0006 |
| 20:5 | 0 vs 1000 | 0.04267 | **** | <0.0001 |
|  | 0 vs 2000 | 0.05833 | **** | <0.0001 |
|  | 0 vs 4000 | 0.08600 | **** | <0.0001 |
|  | 1000 vs 2000 | 0.01567 | ** | 0.0023 |
|  | 1000 vs 4000 | 0.04333 | **** | <0.0001 |
|  | 2000 vs 4000 | 0.02767 | **** | <0.0001 |
| 5:0 | 0 vs 1000 | 0.003333 | ns | 0.8581 |
|  | 0 vs 2000 | 0.005000 | ns | 0.6372 |
|  | 0 vs 4000 | 0.007333 | ns | 0.3114 |
|  | 1000 vs 2000 | 0.001667 | ns | 0.9788 |
|  | 1000 vs 4000 | 0.004000 | ns | 0.7782 |
|  | 2000 vs 4000 | 0.002333 | ns | 0.9451 |
| 5:1.25 | 0 vs 1000 | 0.007333 | ns | 0.3114 |
|  | 0 vs 2000 | 0.01433 | ** | 0.0062 |
|  | 0 vs 4000 | 0.02533 | **** | <0.0001 |
|  | 1000 vs 2000 | 0.007000 | ns | 0.3522 |
|  | 1000 vs 4000 | 0.01800 | *** | 0.0004 |
|  | 2000 vs 4000 | 0.01100 | ns | 0.0533 |
| 5:10 | 0 vs 1000 | 0.02633 | **** | <0.0001 |
|  | 0 vs 2000 | 0.05333 | **** | <0.0001 |
|  | 0 vs 4000 | 0.09967 | **** | <0.0001 |
|  | 1000 vs 2000 | 0.02700 | **** | <0.0001 |
|  | 1000 vs 4000 | 0.07333 | **** | <0.0001 |
|  | 2000 vs 4000 | 0.04633 | **** | <0.0001 |
|  |  |  |  |  |
| BPD (µM): ICG (µM) | 800 nm  pulsed laser | Mean Difference | Summary | Adjusted  p-value |
| 0:5 | 0 vs 1000 | 0.004000 | ns | 0.4945 |
|  | 0 vs 2000 | 0.007333 | ns | 0.0555 |
|  | 0 vs 4000 | 0.01233 | *** | 0.0003 |
|  | 1000 vs 2000 | 0.003333 | ns | 0.6419 |
|  | 1000 vs 4000 | 0.008333 | * | 0.0225 |
|  | 2000 vs 4000 | 0.005000 | ns | 0.2975 |
| 1.25:5 | 0 vs 1000 | 0.004000 | ns | 0.4945 |
|  | 0 vs 2000 | 0.008333 | * | 0.0225 |
|  | 0 vs 4000 | 0.01633 | **** | <0.0001 |
|  | 1000 vs 2000 | 0.004333 | ns | 0.4239 |
|  | 1000 vs 4000 | 0.01233 | *** | 0.0003 |
|  | 2000 vs 4000 | 0.008000 | * | 0.0307 |
| 5:5 | 0 vs 1000 | 0.005667 | ns | 0.1968 |
|  | 0 vs 2000 | 0.001000 | ns | 0.9847 |
|  | 0 vs 4000 | 0.01367 | **** | <0.0001 |
|  | 1000 vs 2000 | -0.004667 | ns | 0.3578 |
|  | 1000 vs 4000 | 0.008000 | * | 0.0307 |
|  | 2000 vs 4000 | 0.01267 | *** | 0.0002 |
| 10:5 | 0 vs 1000 | 0.003333 | ns | 0.6419 |
|  | 0 vs 2000 | 0.006000 | ns | 0.1568 |
|  | 0 vs 4000 | 0.01133 | *** | 0.0009 |
|  | 1000 vs 2000 | 0.002667 | ns | 0.7815 |
|  | 1000 vs 4000 | 0.008000 | * | 0.0307 |
|  | 2000 vs 4000 | 0.005333 | ns | 0.2437 |
| 20:5 | 0 vs 1000 | 0.001667 | ns | 0.9348 |
|  | 0 vs 2000 | 0.005333 | ns | 0.2437 |
|  | 0 vs 4000 | 0.01033 | ** | 0.0028 |
|  | 1000 vs 2000 | 0.003667 | ns | 0.5679 |
|  | 1000 vs 4000 | 0.008667 | * | 0.0163 |
|  | 2000 vs 4000 | 0.005000 | ns | 0.2975 |
| 5:0 | 0 vs 1000 | 0.001333 | ns | 0.9650 |
|  | 0 vs 2000 | 0.0003333 | ns | 0.9994 |
|  | 0 vs 4000 | 0.001667 | ns | 0.9348 |
|  | 1000 vs 2000 | -0.001000 | ns | 0.9847 |
|  | 1000 vs 4000 | 0.0003333 | ns | 0.9994 |
|  | 2000 vs 4000 | 0.001333 | ns | 0.9650 |
| 5:1.25 | 0 vs 1000 | 0.0003333 | ns | 0.9994 |
|  | 0 vs 2000 | 0.000 | ns | >0.9999 |
|  | 0 vs 4000 | 0.003000 | ns | 0.7139 |
|  | 1000 vs 2000 | -0.0003333 | ns | 0.9994 |
|  | 1000 vs 4000 | 0.002667 | ns | 0.7815 |
|  | 2000 vs 4000 | 0.003000 | ns | 0.7139 |
| 5:10 | 0 vs 1000 | 0.003333 | ns | 0.6419 |
|  | 0 vs 2000 | 0.005667 | ns | 0.1968 |
|  | 0 vs 4000 | 0.01867 | **** | <0.0001 |
|  | 1000 vs 2000 | 0.002333 | ns | 0.8421 |
|  | 1000 vs 4000 | 0.01533 | **** | <0.0001 |
|  | 2000 vs 4000 | 0.01300 | *** | 0.0001 |
